# Loss of PMR4 callose synthase triggers jasmonic acid–dependent resistance to the clubroot disease in *Arabidopsis* and *Brassica napus*

**DOI:** 10.1093/plcell/koag160

**Published:** 2026-06-03

**Authors:** Runbang Luo, Lipu Wang, Rui Wen, Kun Yang, Xunjia Liu, Peng Gao, Jiangying Tu, Tim Dumonceaux, Yangdou Wei, Gary Peng, Wei Xiao

**Affiliations:** Department of Biochemistry, Microbiology and Immunology, University of Saskatchewan, Saskatoon, SK, Canada S7N 5E5; Department of Biochemistry, Microbiology and Immunology, University of Saskatchewan, Saskatoon, SK, Canada S7N 5E5; Department of Plant Sciences, University of Saskatchewan, Saskatoon, SK, Canada S7N 5A8; Agriculture and Agri-Food Canada, Saskatoon Research and Development Centre, Saskatoon, SK, Canada S7N 0X2; Department of Biochemistry, Microbiology and Immunology, University of Saskatchewan, Saskatoon, SK, Canada S7N 5E5; Agriculture and Agri-Food Canada, Saskatoon Research and Development Centre, Saskatoon, SK, Canada S7N 0X2; Agriculture and Agri-Food Canada, Saskatoon Research and Development Centre, Saskatoon, SK, Canada S7N 0X2; Agriculture and Agri-Food Canada, Saskatoon Research and Development Centre, Saskatoon, SK, Canada S7N 0X2; Agriculture and Agri-Food Canada, Saskatoon Research and Development Centre, Saskatoon, SK, Canada S7N 0X2; Department of Biology, University of Saskatchewan, Saskatoon, SK, Canada S7N 5E2; Agriculture and Agri-Food Canada, Saskatoon Research and Development Centre, Saskatoon, SK, Canada S7N 0X2; Department of Biochemistry, Microbiology and Immunology, University of Saskatchewan, Saskatoon, SK, Canada S7N 5E5

## Abstract

Clubroot caused by the protist pathogen *Plasmodiophora brassicae* Woronin is a major disease in Brassicaceae crops. Growing clubroot-resistant cultivars remains the most effective and practical control strategy, but diverse or emerging pathotypes can often overcome resistance genes deployed. In this study, we identified *POWDERY MILDEW RESISTANT 4* (*PMR4*) as a potential host susceptibility factor that can be targeted as a novel genetic resource for durable clubroot resistance. Recessive *PMR4* mutations in *Arabidopsis thaliana* conferred strong resistance to multiple *P. brassicae* pathotypes, independent of salicylic acid–mediated plant immunity. Using CRISPR/Cas9, we edited 2 *PMR4* orthologs in the *Brassica napus* genome, and the resulting homozygous mutants exhibited resistance to both powdery mildew and clubroot diseases. This study reveals that *P. brassicae* infection induces callose deposition in *Arabidopsis* and *B. napus* roots in a PMR4-dependent manner, similar to wound- and powdery mildew–induced callose deposition in *Arabidopsis* leaves. The *Arabidopsis pmr4-1* mutation does not affect *P. brassicae* primary infection but blocks the pathogen entry from epidermal cells to the stele in a jasmonic acid signaling–dependent manner, which coincides with accumulation of lignin-like and suberin compounds in periderm cell walls. Together, these findings establish *PMR4*-encoded callose synthase as a host susceptibility factor facilitating secondary infection by *P. brassicae* and demonstrate its potential as a gene-editing target for enhancing resistance to powdery mildew, clubroot, and possibly other biotic and abiotic stresses in Brassicaceae crops.

## Introduction

Brassicaceae crops, especially canola/oilseed rape (*Brassica napus* L.), are cultivated globally with significant economic and nutritional values. However, most of these crops are affected by clubroot, a severe soil-borne disease caused by a protist *Plasmodiophora brassicae* (*Pb*) Woronin (Cardoza and Stewart 2006; Pérez-López et al. 2018). Resting spores, as the primary inoculum of the pathogen, can survive for up to 17 yr in the soil (Wallenhammar 1996). Primary zoospores released from resting spores initially infect root hairs, producing primary plasmodia during this infection stage that do not cause macroscopic symptoms (Howard et al. 2010; Hwang et al. 2012). The secondary zoospores released from root hairs can penetrate root epidermis and invade cortical and inner tissues of main roots, resulting in club-shaped root galls (Fig. S1) (Hwang et al. 2012; Liu et al. 2020a). The gall formation impairs water and nutrient uptake, leading to plant wilting and premature death (Hwang et al. 2012; Schwelm et al. 2015).

The most efficient disease management for clubroot to date is growing clubroot-resistant (*CR*) cultivars in various rotations (Hwang et al. 2012; Peng et al. 2015). Although there are several commercial *CR* oilseed and cabbage cultivars available, their resistance is controlled by a single limited number of dominant *CR* genes, which can be broken quickly (Matsumoto et al. 2012), especially given the diverse and evolving pathogen populations found in western Canada (Strelkov et al. 2018). Distinct from the *R*-gene strategy, an emerging alternative approach based on susceptibility (*S*) genes has been gaining interest due to its potential to be more durable than the conventional *R-*gene-driven disease resistance in the field (Zaidi et al. 2018). These *S* genes may facilitate host–pathogen compatibility by promoting one or more steps of the infection process (van Schie and Takken 2014). Increasing evidence has shown promise to create such novel disease-resistant crops through the alteration of *S* genes. With the development of genome-editing techniques like CRISPR/Cas9 in plants (Doudna and Charpentier 2014; Gao 2021), the target *S* genes can be precisely modified, leading to transgene-free crops (Soda et al. 2018; Boubakri 2023) with potentially durable resistance to plant diseases.

We reasoned that *Arabidopsis thaliana* serves as an ideal model system for screening and identification of clubroot *S* (*CS*) genes. Firstly, *A. thaliana* is genetically and physiologically related to the rapeseed species. Secondly, the *Arabidopsis* ecotype Col-0 is susceptible to all known *Pb* pathotypes. Thirdly, many T-DNA insertion and mutagen-induced mutants in Col-0 are available in the public domain, making the screening of diverse genotypes possible. In this study, we screened a range of mutants and identified *POWDERY MILDEW RESISTANT 4* (*PMR4*) as a promising *CS* gene. *PMR4* encodes a putative callose synthase (Vogel and Somerville 2000) and was previously identified as an *S* gene in the context of powdery mildew (PM) disease on *Arabidopsis* because the loss of PMR4 callose synthase activities resulted in salicylic acid (SA)–dependent plant defense responses against PM (Vogel and Somerville 2000; Nishimura et al. 2003). Unexpectedly, our experimental data showed that disrupting the SA signaling in the *pmr4* mutant did not restore its susceptibility to clubroot disease; instead, *pmr4*-conferred clubroot resistance was dependent on jasmonic acid (JA) signaling and correlated with the biosynthesis of lignin-like and suberin compounds in root periderm cell walls. These findings agree with a recent report (Wu et al. 2025). Targeted genome editing of *B. napus PMR4* orthologs confers dual resistance to both PM and clubroot, highlighting its potential for novel disease resistance in Brassicaceae crops.

## Results

### *Arabidopsis pmr4-1* mutant confers strong resistance against multiple *Pb* pathotypes

**Figure 1:**
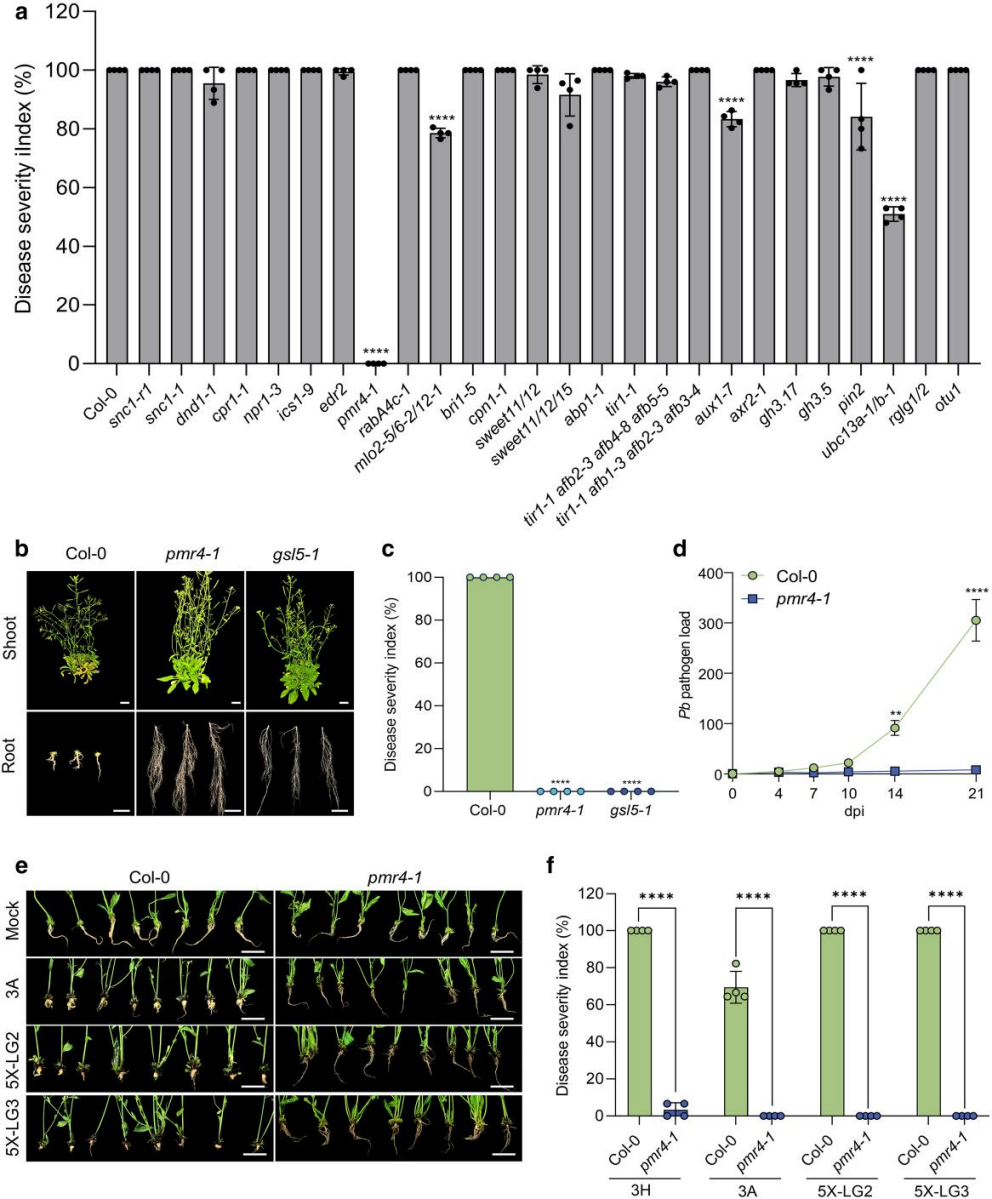
*Arabidopsis pmr4-1* and *gsl5-1* are highly resistant to *Pb*. a) Pathogenicity assay on selected *Arabidopsis* mutants against the clubroot disease. DSI reflects quantitative assessments to the disease. Clubroot symptoms were rated at 21 dpi after *Pb* pathotype 3H inoculation for at least 4 biological replicates with each line containing 15 plants. Data are means ± Sd obtained from 4 independent experiments. Statistical differences were analyzed by 1-way ANOVA taking Col-0 as a control. ****P ≤ 0.0001. b) Representative shoot (upper row) and root (lower row) images of *Pb* pathotype 3H-inoculated wild-type (Col-0), *pmr4-1* and *gsl5-1* mutants captured at 21 dpi. Scale bar = 2 cm. c) Quantitative analysis of *Arabidopsis* clubroot disease severity as shown in b). DSI values are means ± Sd obtained from 4 biological repeats with 15 plants for each replicate. ****P ≤ 0.0001. d) Relative *Pb* pathogen biomass by quantitative PCR as described (Wen et al. 2020). *AtUBQ10* gene was used as host reference. Values are relative to the inoculated plants at 0 dpi. At least 5 plant roots were pooled as 1 biological replicate. Data represent means ± Sd. *n* = 3. Two-way ANOVA analysis for relative *Pb* biomass was performed to compare the pathogen load differences between Col-0 and *pmr4-1*, and asterisk(s) represent statistical significance (α = 0.05). **P ≤ 0.01; ****P ≤ 0.0001. e) Representative root images of Col-0 and *pmr4-1* inoculated with various *Pb* pathotypes captured at 21 dpi. Scale bar = 2 cm. f) DSI (means ± Sd) of Col-0 and *pmr4-1* inoculated with various *Pb* pathotypes. *n* = 4. ****P ≤ 0.0001.

To identify *CS* genes, *Arabidopsis* mutants carrying reported *S* genes that conferred enhanced resistance against biotrophic/hemi-biotrophic pathogens were collected. These mutant plants were inoculated with the *Pb* field isolate of pathotype 3H, the most prevalent pathotype in western Canada with high virulence (Hollman et al. 2023), followed by the assessment of severity against clubroot infection at 21 d postinoculation (dpi). Among 26 mutant lines tested to date (Table S1), a *mlo2,6,12* triple mutant and a *ubc13a,b* double mutant displayed moderate resistance to clubroot (Fig. 1a). *MILDEW RESISTANCE LOCUS O* (*MLO*) genes encode plant-specific calcium channels (Gao et al. 2022), and loss of *MLO* family genes confers resistance to fungal PM in the model plant *Arabidopsis* (Consonni et al. 2006) and crop species including barley, wheat, pea, and tomato (Jørgensen 1992; Kusch and Panstruga 2017). *UBC13s* encode ubiquitin conjugation enzymes that mediate noncanonical K63-linked polyubiquitination (Hofmann and Pickart 1999; McKenna et al. 2001). The *Arabidopsis ubc13a,b* double mutation compromises auxin signaling, root growth (Wen et al. 2014), and immune responses (Wang et al. 2019). Surprisingly, a *sweet11,12* double mutant, which was reported to be clubroot resistant (Walerowski et al. 2018), and a corresponding *sweet11,12,15* triple mutant did not display an apparent reduction in the disease severity index (DSI) under our experimental conditions (Fig. 1a). In sharp contrast, the *pmr4-1* mutant consistently displayed strong resistance to clubroot with DSI close to 0% and almost no visible gall formation across 6 independent experiments (Fig. 1a).

*PMR4*, also known as *GSL5* and *CalS12*, encodes a predicted trans-membrane callose synthase (Zaveská Drábková and Honys 2017). *PMR4* was initially identified because its mutant alleles conferred PM resistance (Vogel and Somerville 2000). The *Arabidopsis pmr4-1* mutation contains a single G-to-A base substitution in the second exon of the *PMR4* gene, resulting in the conversion of codon TGG for Trp-687 to a stop codon TAG and the formation of a prematurely truncated polypeptide of 686 amino acids in length (Nishimura et al. 2003). This truncated PMR4 variant retains the N-terminal 6 transmembrane helices but lacks the putative catalytic domain and the subsequent 7 transmembrane helices at the C terminus. To independently assess whether the enhanced clubroot resistance is caused by the nonsense mutation in *PMR4,* a T-DNA insertion line *gsl5-1* (GABI-KAT 089H05), in which a T-DNA is inserted to the second exon of *PMR4* close to the nonsense mutation in *pmr4-1* (see Fig. S8a), was acquired and inoculated with *Pb* pathotype 3H. Typical swollen root galls were observed in wild-type roots, and the plants were severely affected at 21 dpi, while *pmr4-1* and *gsl5-1* plants remained healthy without visible symptoms both underground and aboveground (Fig. 1b), with DSI values near 0% (Fig. 1c). Similarly, *Pb* biomass in Col-0 increased steadily from 10 to 21 dpi, while no measurable increase was observed in *Pb*-infected *pmr4-1* during this period (Fig. 1d). This result indicates that the enhanced clubroot resistance observed in *pmr4-1* and *gsl5-1* mutants is exclusively caused by mutations in the *PMR4* gene.

Since both *pmr4-1* and *gsl5-1* can potentially produce truncated proteins, it is unclear whether they are complete loss-of-function mutations. To determine the nature of mutations for clubroot resistance, a heterozygous *gsl5-1* line and its self-pollinated segregants were assessed by *Pb* inoculation as above. The *gsl5-1*/+ heterozygous plants were largely susceptible to the clubroot pathogen, and their self-pollinated progenies segregated into 16 resistant and 43 susceptible individuals. A chi-square test showed that it fits a phenotypic segregation ratio of 3:1 (χ^2^_3:1_ = 0.142), indicating that the *gsl5-1*-mediated clubroot resistance is recessively inherited. Hence, we conclude that the enhanced clubroot resistance is conferred by a recessive mutation at the *PMR4/GSL5* locus.

The involvement of *PMR4* in clubroot susceptibility encouraged us to further determine if the resistance observed in the *pmr4*-*1* mutant is race/pathotype dependent. In addition to pathotype 3H, *pmr4-1* mutant plants were also highly resistant to 3 additional pathotypes/isolates found in western Canada, including 3A, 5X-LG2, and 5X-LG3 (Fig. 1e and f). Hence, the loss of *PMR4/GSL5* resulted in robust clubroot resistance against multiple *P. brassica* pathotypes under our experimental conditions.

### *pmr4-1*-conferred clubroot resistance is independent of SA signaling

*pmr4-1* has been extensively studied for its resistance to the fungal disease PM (Vogel and Somerville 2000; Jacobs et al. 2003; Nishimura et al. 2003). A widely accepted explanation for this resistance is that callose or the callose synthase encoded by *PMR4* negatively regulates the SA pathway–mediated immunity. Consequently, PM resistance in the *pmr4-1* mutant is attributed to enhanced activation of the SA signaling pathway (Nishimura et al. 2003). Given the substantial biological and ecological differences between this foliar fungal pathogen and the root-infecting protist that causes clubroot, we investigated whether the enhanced clubroot resistance in the *pmr4-1* mutant is also dependent on the functional SA pathway.

**Figure 2:**
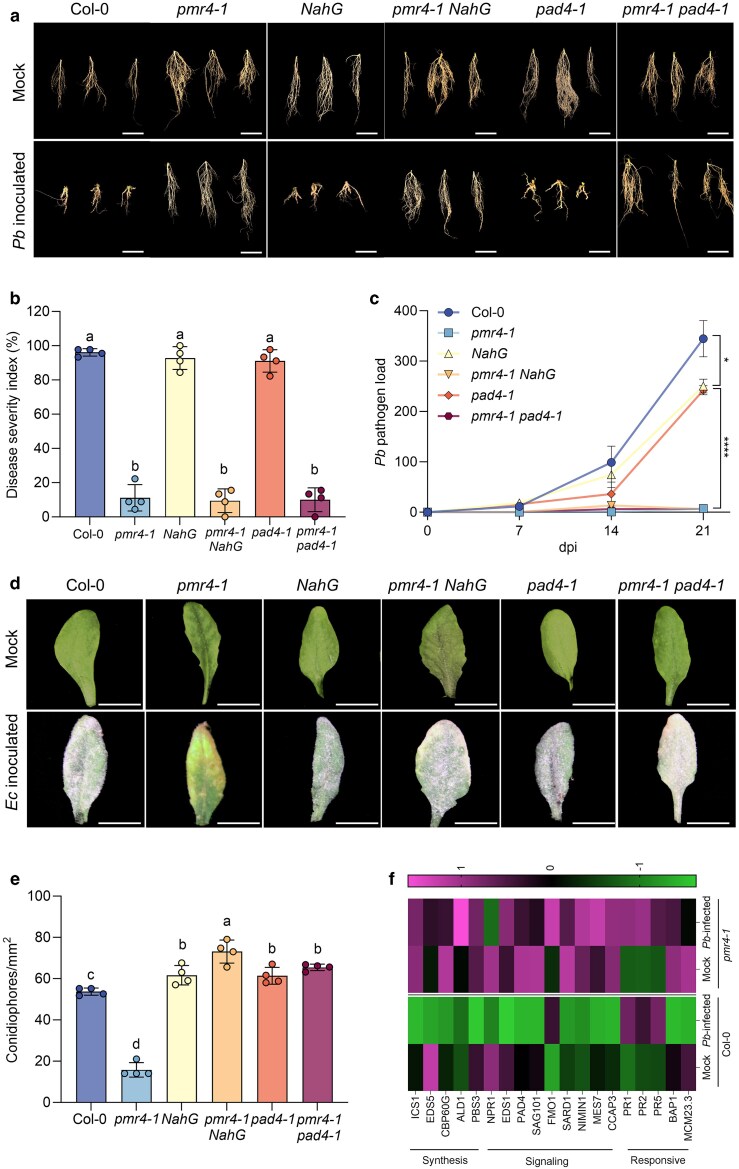
*Arabidopsis pmr4-1* confers dual resistance to *Pb* and *Ec*. a) Representative root images of plants from indicated genotypes with and without *Pb* pathotype 3H inoculation at 21 dpi. Scale bar = 2 cm. b) Quantitative analysis of *Arabidopsis* clubroot disease severity as shown in a). DSI values are means ± Sd obtained from 3 groups of biological repeats, each containing 15 individual plants. One-way ANOVA was performed, and different letters represent various disease severity levels as well as statistical significance. c) Relative *Pb* biomass as measured by qPCR of a *Pb* internal transcribed spacer 1 region (Wen et al. 2020). *AtUBQ10* was used as a host tissue normalization reference. At least 3 plant roots were pooled to form 1 mixed sample with 3 biological replicates for each line. Data are means ± Sd obtained from 3 technical replicates. One-way ANOVA analysis for *Pb* relative biomass at 21 dpi was performed. *P ≤ 0.05, ****P ≤ 0.0001. d) Representative leaf images of plants from indicated genotypes with and without *Ec* inoculation at 15 dpi. Scale bar = 2 cm. e) Quantitative analysis of *Ec* conidiophore density on *Ec*-inoculated leaves of indicated genotypes at 5 dpi. One-way ANOVA was performed, and different letters represent statistical significance. Data represent means ± Sd. f) Transcriptome profiling of the gene expressions involved in SA and N-hydroxypipecolic acid biosynthesis, signaling, and response in the roots before or after *Pb* inoculation at 14 dpi. Heatmap was generated based on the *Z*-score from 3 biological repeats for each group of samples.

To determine whether *pmr4-1*-mediated clubroot resistance depends on the SA pathway, we tested a *pmr4-1 pad4-1* double mutant and a transgenic *pmr4-1 NahG* line, both with compromised SA signaling, for response to the clubroot infection. PAD4 acts upstream of SA accumulation in a positive feedback loop required for defense activation (Zhou et al. 1998; Jirage et al. 1999), while bacterium-derived enzyme NahG specifically degrades SA (Delaney et al. 1994). These lines have been previously used to demonstrate the SA dependency of *pmr4-1*-mediated PM resistance (Nishimura et al. 2003). To our surprise, both *pmr4-1 pad4-1* and *pmr4-1 NahG* plants remained resistant to clubroot, exhibiting phenotypes indistinguishable from those of *pmr4-1* single mutant (Fig. 2a). In contrast, severe galling was observed on Col-0, *NahG*, and *pad4-1* plants following *Pb* inoculation (Fig. 2a). Clubroot DSI (Fig. 2b) further confirmed that blocking the SA signaling pathway did not compromise *pmr4*-conferred clubroot resistance.

To avoid bias in the disease severity assessment, we performed quantitative real-time PCR (qPCR) to monitor pathogen biomass in infected root tissues postinoculation. In agreement with the disease severity result, *Pb* biomass was barely detectable in all *pmr4* mutant backgrounds at all time points examined (Fig. 2c), indicating that the SA signaling is not required for *pmr4*-mediated clubroot resistance. Interestingly, *NahG* and *pad4-1* roots showed lower *Pb* biomass than Col-0 (Fig. 2c), indicating that SA signaling could enhance *Pb* susceptibility.

To test whether SA independence of *pmr4-1*-mediated clubroot resistance is *Pb* pathotype specific, we inoculated wild-type, *pmr4-1*, *pmr4-1 pad4-1*, and *pmr4-1 NahG* plants with *Pb* pathotypes/isolates 3A, 5X-LG2, and 5X-LG3. Resistance in both *pmr4-1 pad4-1* and *pmr4-1 NahG* plants was consistent across all pathotypes/isolates and comparable to *pmr4-1*, while Col-0 and all SA-deficient single mutants remained susceptible (Fig. S2).

To further address the distinct requirements of SA defense pathway in *pmr4-1* and determine whether *pmr4-1* can confer dual resistance against PM and clubroot diseases, we simultaneously inoculated the same plants with conidiospores of the host-adapted *Erysiphe cruciferarum* (*Ec*) on the foliage and *Pb* resting spores in soil. As expected, typical PM symptoms were observed in Col-0, *NahG*, *pad4-1*, *pmr4-1 NahG*, and *pmr4-1 pad4-1*. In contrast, only *pmr4-1* showed leaf lesion spots (Fig. 2d), indicative of SA-mediated hypersensitive reaction. In addition, parasitic structures of *Ec* were monitored by staining inoculated leaf samples with acidic aniline blue staining to *Ec*-inoculated leaf samples, which revealed enhanced resistance to *Ec* by the *pmr4-1* mutation, as evidenced by decreased hyphal growth rate of *Ec* with fewer hyphal branches and a lower density than Col-0 in conidiophores and disease symptoms on the leaf surface at 7 dpi in *pmr4-1* plants (Fig. S3). Furthermore, inactivation of the SA signaling pathway in *pmr4-1* mutants led to increased conidiophore formation in *Ec*-inoculated leaves (Fig. 2e), confirming that SA is required for *pmr4-1*-mediated PM resistance. Taken together, we conclude that *pmr4-1* exhibited dual resistance to PM and clubroot diseases in SA-dependent and SA-independent manners, respectively.

To ensure that the SA pathway was indeed inactivated in *pad4-1* and *NahG* transgenic lines, we examined expression levels of the SA signaling marker gene *PR1* in root tissues of above genotypes following *Pb* inoculation. Consistent with previous reports (Chen et al. 2016; Lovelock et al. 2016), *PR1* was strongly induced in Col-0 but not in *pmr4-1* roots at 21 dpi, while in early time points, *PR1* expression in response to the *Pb* infection was delayed and then diminished in *pmr4-1* roots (Fig. S4a). Furthermore, *pad4-1* significantly reduced and *NahG* abolished *PR1* expression in response to the *Pb* infection (Fig. S4a). These observations collectively confirmed that the *pad4-1* mutation and *NahG* transgene blocked the SA signaling.

To investigate the influence of *Pb* infection on the entire SA pathway, we examined the expression of genes involved in SA biosynthesis, signaling, and responses in root tissues of Col-0 and *pmr4-1* with or without *Pb* inoculation by RNA-seq. The results showed that the SA pathway genes in Col-0 roots were largely suppressed by *Pb* infection at 14 dpi, consistent with previous reports (Ludwig-Müller et al. 2015; Bulman et al. 2019; Djavaheri et al. 2019), while *FMO1*, *PR1*, *PR2*, and *PR5* were slightly induced (Fig. 2f). In contrast, the transcript levels of many SA pathway genes increased moderately in the corresponding *pmr4-1* roots, and they were not suppressed by *Pb* infection (Fig. 2f). These RNA-seq data were further supported by reverse transcription quantitative PCR (RT-qPCR) analysis of selected genes (Fig. S4b to g). Together with our phenotypic analyses, we conclude that although the SA pathway activity is not suppressed in *pmr4-1* roots upon *Pb* infection, the *pmr4-*conferred clubroot resistance does not require functional SA signaling.

### Genome-edited *B. napus PMR4* mutants are dually resistant to clubroot and PM diseases

The *pmr4-1* mutation did not show significant impact on *Arabidopsis* plant growth and development (Fig. S5), making *PMR4* an ideal target for engineering clubroot resistance. Many crops belonging to the Brassicaceae family suffer from the clubroot disease (Hwang et al. 2012). Since loss of the PMR4 activity in *Arabidopsis* confers clubroot resistance, we investigated whether the *PMR4* gene is conserved among these crops and whether targeting *PMR4* orthologs in *B. napus* also leads to resistance to clubroot. The *Arabidopsis* PMR4 amino acid sequence was used to blast the Ensembl Plants database (https://plants.ensembl.org/Multi/Tools/Blast) focusing on Brassicaceae crops with high economic values. A phylogenetic analysis of candidate *PMR4* orthologs (Fig. S6) shows that these genes are highly conserved and widely present across *Brassicaceae* species. Two genes in the *B. napus* genome, annotated as *BnaC09g00800D* (*BnaPMR4.C09*) and *BnaA09g01630D* (*BnaPMR4.A09*), encode proteins with 91% amino acid sequence identity to *Arabidopsis* PMR4 (*E*-values at or below 10^−56^) (Fig. S7). Similar to AtPMR4, the predicted BnaPMR4.C09 and BnaPMR4.A09 are polytopic integral membrane proteins with 10 and 12 transmembrane helices, respectively (Fig. S8) and contain 2 conserved potential catalytic domains: 1,3-β-glucan synthase component FKS1-like domain (FKS1_dom1) and 1,3-β-glucan synthase domain (Hsieh et al. 2024).

**Figure 3:**
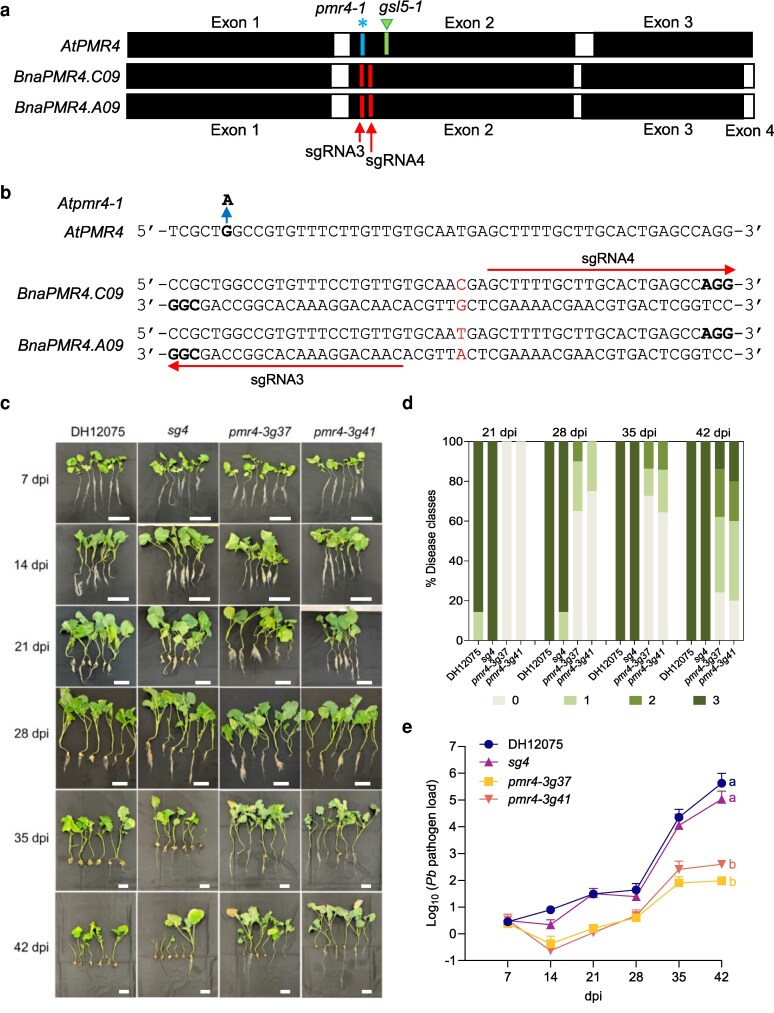
Creation and clubroot disease test of *B. napus pmr4* mutants. a) Comparison of predicted *Arabidopsis* and *B. napus PMR4* genes. Filled and open boxes indicate exons and introns, respectively. Blue asterisk indicates the point mutation site in *pmr4-1*; green triangle indicates the T-DNA insertion site in *gsl5-1*; red arrows indicate target sites of sgRNAs for *BnaPMR4* genes. b) *B. napus* sequences to show sgRNA target sites. PAM sequences are in bold. c) Representative root and shoot phenotypes of *B. napus* plants infected with *Pb* pathotype 3H. Scale bar = 5 cm. d) The clubroot disease symptom as shown in c) was recorded by a 0 to 3 class-scoring system. Values were obtained from at least 10 to 30 individual roots per genotype at each time point. Distinct colors indicate the proportions of different disease classes. e) *Pb* pathogen load quantified by ddPCR targeting on a segment of the internal transcribed spacer 1 region of *Pb* relative to *B. napus* housekeeping gene *BnActin*. Plants were inoculated with 1 × 10^8^ spore/mL *Pb* pathotype 3H resting spore inoculum and were assessed at the indicated dpi. Two-way ANOVA was performed, and different letters represent statistical significance (α = 0.05). Data represent means ± Sd. At least 5 individual roots from each genotype were collected as 1 biological repeat. Three repeats were assessed for each treatment.

To create *B. napus PMR4* mutants that mimic *Arabidopsis pmr4-1* and *gsl5-1*, 2 sgRNA constructs in Exon 2 capable of targeting both *PMR4* genes (Fig. 3a and b) were designed and separately assembled into a CRISPR/Cas9 vector. These CRISPR/Cas9 sgRNA constructs were transformed into a *B. napus* doubled haploid line DH12075 by using Agrobacterium-mediated hypocotyl transformation (Zhou et al. 2002). While no mutation was yet found in sgRNA4 transformed plants, 2 sgRNA3 transformed lines were found to carry +1 frameshift mutations in both target genes. Line *pmr4*-*3g37* harbored biallelic homozygous (T/T) insertions in *BnaPMR4.C09* (Fig. S9a) and biallelic heterozygous (T/G) insertions in *BnaPMR4.A09* (Fig. S9b), making it a T0 homozygous double mutant. On the other hand, line *pmr4*-*3g41* carried monoallelic (T/−) insertions in both *BnPMR4* genes (Fig. S9a and b), and was therefore a T0 heterozygous double mutant. The gene-edited homozygous *pmr4*-*3g37* (T3 generation) and *pmr4-3g41* (T2 generation) lines were evaluated for the clubroot resistance against *Pb* pathotype 3H alongside reference lines including DH12075 (parental) and *sg4* (an sgRNA4 transgenic DH12075 line with unedited *PMR4* genes). *Pb*-infected *pmr4*-*3g37* and *pmr4-3g41* plants remained healthy, displaying no aboveground symptoms such as chlorosis or stunting (Fig. 3c). Furthermore, no visible root swelling or galling was observed in these lines up to 28 dpi. At 35 dpi and beyond, these 2 *B. napus pmr4* mutants formed much smaller galls and only portion of their roots compared to the susceptible lines, which formed large root galls on the main root of all plants (Fig. 3c). Importantly, the aboveground portion of *Pb*-infected *pmr4-3g37* and *pmr4-3g41* plants grew normally even at 42 dpi, whereas DH12075 and *sg4* plants displayed stunted growth (Fig. 3c) and eventually died. Disease severity ratings (Fig. 3d) supported the observed resistance phenotypes. Additionally, droplet digital PCR (ddPCR) analysis showed significantly lower *Pb* biomass in the infected roots of *pmr4-3g37* and *pmr4-3g41* compared to DH12075 and *sg4* (Fig. 3e). Similar resistance was observed against *Pb* pathotypes 3A and 5X-LG2, with *pmr4-3g37* displaying resistance comparable to a *CR* control NAS6. In contrast, susceptible controls Westar (susceptible commercial line), DH12075 (parental) and 45H29 (a formerly resistant cultivar subsequently overcome by newer *Pb* pathotypes) (Jiang et al. 2020) were all susceptible under the same experimental conditions (Fig. S10a).

The T0 heterozygous *pmr4*-*3g41* line was self-pollinated, and the T1 segregants were tested for resistance against the newly-reported *Pb* pathotype 3A. Out of a population of 141 plants assessed, 10 were resistant while 131 were susceptible (Fig. S10b), which would fit well with the expected 2-gene segregation ratio of 15:1, provided both mutations are recessive. All 10 resistant plants contained homozygous mutations in both *PMR4* genes. Similarly, when *pmr4*-*3g41* T1 segregants were inoculated with *Pb* pathotype 3H, 8 out of 107 plants were resistant (Fig. S10c), and all resistant plants carried homozygous mutations in both *PMR4* genes. These results demonstrate that *B. napus* homozygous *PMR4* mutants are resistant to multiple *Pb* pathotypes and that both *BnaPMR4s* function as *CS* genes.

**Figure 4:**
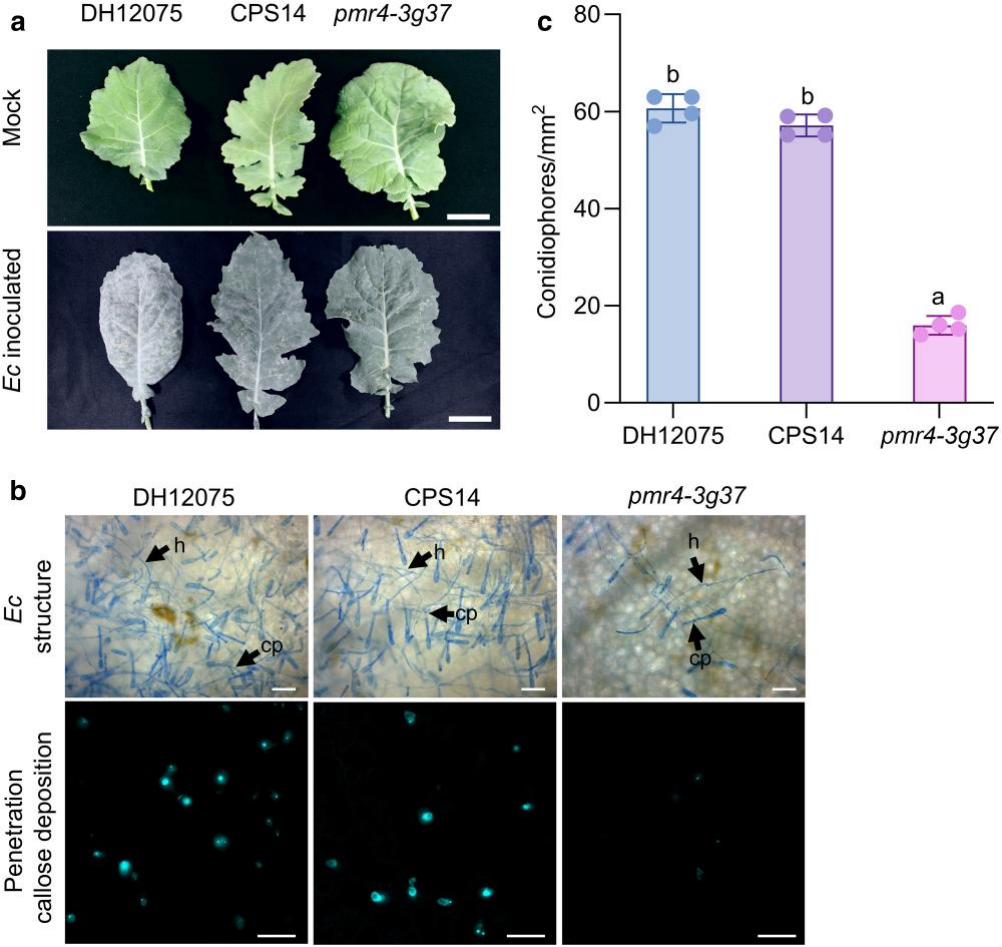
Susceptibility of *B. napus* plants to the PM disease. a) Leaf phenotypes of DH12075, CPS14, and *pmr4-3g37* at 40 d after *Ec* inoculation (lower row). Corresponding uninfected controls are also shown in the upper row. Scale bar = 4 cm. b) Upper row: representative images of hyphae growth and conidiophore production of *Ec* on plant leaf surfaces at 7 dpi. Fungal structures were stained with acidic aniline blue and examined by light microscopy. h, hyphae; cp, conidiophores. Scale bar = 100 μm. Lower row: callose deposition at the *Ec*-penetration sites stained with basic aniline blue and revealed with epifluorescence microscopy. Scale bar = 50 μm. c) Quantitative measurement of *Ec* conidiophore formation on *Ec*-inoculated leaves at 5 dpi. Data are means ± Sd from 30 colonies collected from 3 leaves. One-way ANOVA was performed, and different letters represent statistical significance (α = 0.05).

We also inoculated *pmr4-3g37* plants with host-adapted *Ec* to determine whether *B. napus pmr4* mutants are also resistant to PM. Indeed, *pmr4-3g37* plants exhibited strong resistance to PM as canonical whitish powdery symptoms can be observed on parental DH12075 leaves, while no such disease feature was found on *pmr4-3g37* leaves (Fig. 4a). In contrast, a double *CR* gene resistant line CPS14 (Tu et al. 2024) also showed susceptibility to the *Ec* infection (Fig. 4a). Microscopic observations of *Ec* parasitic structures following acidic aniline blue staining revealed fewer *Ec* hyphal branches at 7 dpi on *pmr4-3g37* leaves than on DH12075 leaves (Fig. 4b, upper row). Furthermore, while *Ec*-inoculated DH12075 and CPS14 leaves showed prominent papillae-associated callose deposition at 2 dpi, the response was dramatically reduced in *pmr4-3g37* leaves (Fig. 4b, lower row). This reduction correlated with decreased conidiophore development in *Bnapmr4* mutants (Fig. 4c), consistent with phenotypes reported for the *Atpmr4-1* mutant (Jacobs et al. 2003). These findings support the conclusion that *BnaPMR4* is a promising candidate gene target for creating novel *CR B. napus* lines with the added benefit of PM resistance.

### *Pb* secondary infection is compromised in *pmr4-1*

To understand why *PMR4* inactivation confers resistance to the clubroot disease, we monitored the *Pb* development in Col-0 and *pmr4-1* roots using multiple methods, including confocal laser-scanning microscopy (CLSM) (Liu et al. 2020a), cytological examination based on cross-section (Walerowski et al. 2018), and transmission electron microscopy (TEM), to track primary and secondary infections. Since 7 dpi typically marks the end of primary *Pb* infection, with stele colonization and symptom development beginning around 14 dpi (Liu et al. 2020a), we collected samples at 7, 10, and 14 dpi to time primary, primary-to-secondary and secondary *Pb* infection stages.

The primary infection appeared to be successful and completed in both Col-0 and *pmr4-1* roots, as zoospores were released from zoosporangia in their epidermal cells at 7 dpi observed by CLSM (Fig. S11a; Videos S1 and S2). Zoospore release was assessed at 14 dpi by examining infected lateral roots near the hypocotyl in both Col-0 and *pmr4-1*, which showed comparable levels of zoospore release at the later infection stage (Fig. S11b). Notably, Z-stack videos captured the successful *Pb* secondary plasmodium colonization in the Col-0 cortex adjacent to an empty zoosporangium in the neighboring epidermal cell (Video S3). In contrast, although numerous empty zoosporangia were detected in *pmr4-1* epidermal cells, secondary plasmodium was barely found in the inner cell layers (Video S4), suggesting dramatically reduced secondary infection in *pmr4-1*.

**Figure 5:**
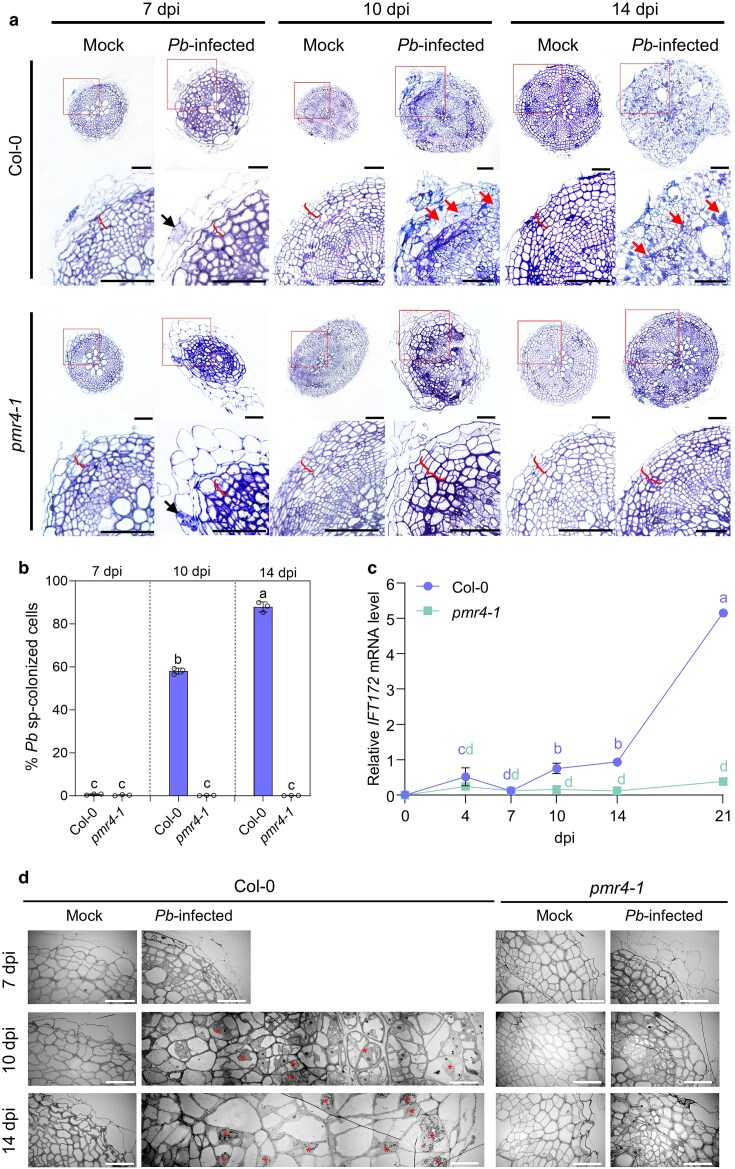
*Pb* disease progression in the Col-0 and *pmr4-1*. a) Representative transverse sections (0.5 *µ*m) of Col-0 and *pmr4-1* roots stained with toluidine blue to show changes in response to the *Pb* infection, with enlarged one-quarter views in corresponding lower rows. Scale bars represent 100 *µ*m. Black and red arrows indicate *Pb* zoosporangium and secondary plasmodium, respectively. Red brackets indicate periderm. b) Proportion of Col-0 and *pmr4-1* cells harboring *Pb* secondary plasmodium (sp). At each time point, 3 independent sections from 3 individual plants were examined to determine secondary infections using Zeiss AxioPlan light microscopy. Data represent means ± Sd. *n* = 3. Two-way ANOVA analysis was performed to compare differences between Col-0 and *pmr4-1* at 7, 10, and 14 dpi (α = 0.05). c) Expression of *Pb* pathogenesis marker genes to quantify secondary infection stages in Col-0 and *pmr4-1*. All data were normalized to the expression of *PbActin1* and relative to the expression level of inoculated plants at 0 dpi. Two-way ANOVA was performed, and different letters represent statistical significance. Data represent means ± Sd. *n* = 3. d) Overview comparisons of Col-0 and *pmr4-1* under TEM before and after *Pb* infection at 7, 10, and 14 dpi under the same magnification from outermost layer to xylem cells. Images of *Pb*-infected Col-0 at 10 and 14 dpi were stitched by serial overlap views due to the root swelling upon infection. Scale bar = 20 *µ*m. Red asterisks indicate the *Pb* secondary plasmodium.

For cross-sectional microscopic analysis of secondary infection, lateral roots from the maturation zone, where pathogen invasion is initiated, were sampled. In *Arabidopsis*, periderm replaces epidermis during secondary growth in the hypocotyl, upper primary root, and old lateral roots, a process initiated by pericycle cell division, followed by endodermal programmed cell death and eventual detachment of epidermal and cortical cells (Wunderling et al. 2018). Consistent with our CLSM observations, comparable number of zoosporangia were found in epidermal cells from both Col-0 and *pmr4-1* at 7 dpi (Fig. 5a), indicating successful primary colonization. In Col-0, cell deformation and swelling were evident by 10 dpi and became more pronounced at 14 dpi, accompanied by increased secondary plasmodia and the absence of a mature periderm (Fig. 5a). During the same period, *Pb*-infected *pmr4-1* roots exhibited no visible cell deformation but instead showed an intensified periderm staining pattern, in which secondary plasmodia were rarely detected (Fig. 5a), suggesting that secondary infection is largely absent in *pmr4-1* roots.

To quantify the establishment of *Pb* secondary infection, we calculated the proportion of host cells colonized by *Pb* secondary plasmodia. As expected, the proportion of colonized cells in Col-0 increased over time, whereas *pmr4-1* roots consistently showed few if any infected cells (Fig. 5b). To further assess the secondary infection phase, we analyzed the expression of a previously characterized secondary phase-specific *Pb* marker gene *intraflagellar transporter 172* (*IFT172*) (Tu, 2018), encoding a putative cilium- and microtubule-associated protein homolog essential for protist flagellar assembly and length maintenance, which was markedly induced in Col-0 upon infection but remained low in *pmr4-1* during the entire infection cycle (Fig. 5c), indicating impaired secondary infection in this mutant.

TEM (Fig. 5d) further revealed that *Pb* growth was arrested in *pmr4-1* root tissues while induced enlarged and deformed cells in Col-0 characteristic of advanced cortical and stele infection. We also measured the average size of healthy and *Pb*-infected root cells from both lines and observed that cell size gradually increased in Col-0 roots at 10 and 14 dpi, corresponding to 20-fold increase in cell volume. In contrast, *pmr4-1* root cells did not change in volume following *Pb* infection (Figs. 5d and  S12a). Meanwhile, *pmr4-1* root periderm cell walls were thicker than Col-0 in the absence of infection and became even thicker after *Pb* infection at 14 dpi (Fig. S12b), whereas cell walls from equivalent Col-0 root layers became thinner following *Pb* infection (Fig. S12b). Together, these observations indicate that loss of PMR4 prevents *Pb* secondary infection and thereby blocks completion of the disease cycle.

### *Pb* infection leads to PMR4-dependent callose deposition in roots

PMR4 is required for wound-induced callose deposition at wound sites (Jacobs et al. 2003; Nishimura et al. 2003), which is thought to mimic plant response to pathogen invasion. A wound-induced callose accumulation assay showed robust callose deposition in Col-0 leaves, whereas this response was absent in *pmr4-1* leaves (Fig. S13a), consistent with previous reports (Jacobs et al. 2003; Nishimura et al. 2003). Col-0 and *pmr4-1* therefore served as positive and negative controls, respectively, for the wound-induced callose deposition response in *B. napus* leaves. Under the same experimental conditions, DH12075 and heterozygous *pmr4-3g41* T0 leaves displayed clear callose deposition at the wound site 24 h after wounding. In contrast, callose accumulation was absent at the wound site in both *pmr4-3g37* and homozygous *pmr4-3g41* T1 mutant leaves (Fig. S13a). Because wound-induced callose deposition remained detectable in heterozygous *pmr4-3g41* leaves, both *Bnapmr4* alleles are considered loss-of-function mutations. These results indicate that PMR4 is required for wound-induced callose deposition in both *Arabidopsis* and *B. napus*.

Considering that PMR4 functions as a callose synthase responsive to fungal pathogens and wounding in leaves (Nishimura et al. 2003), we asked whether the *Pb* infection also induces callose deposition in *B. napus* root tissues and whether PMR4 is required for this process. To test this, DH12075 and *pmr4-3g37* roots were examined by aniline blue staining, with or without *Pb* pathotype 3H inoculation. At 14 dpi, transverse root sections were prepared and stained with aniline blue to visualize callose (Fig. S13b). In uninoculated roots, only background signals, namely phloem callose and xylem lignin autofluorescence, were observed in both DH12075 and *pmr4-3g37*, consistent with previous reports (Truernit et al. 2008; Xie et al. 2011). Following *Pb* inoculation, DH12075 roots showed clear callose accumulation (white arrows), whereas corresponding *pmr4-3g37* root tissues lacked callose deposition (Fig. S13b).

**Figure 6:**
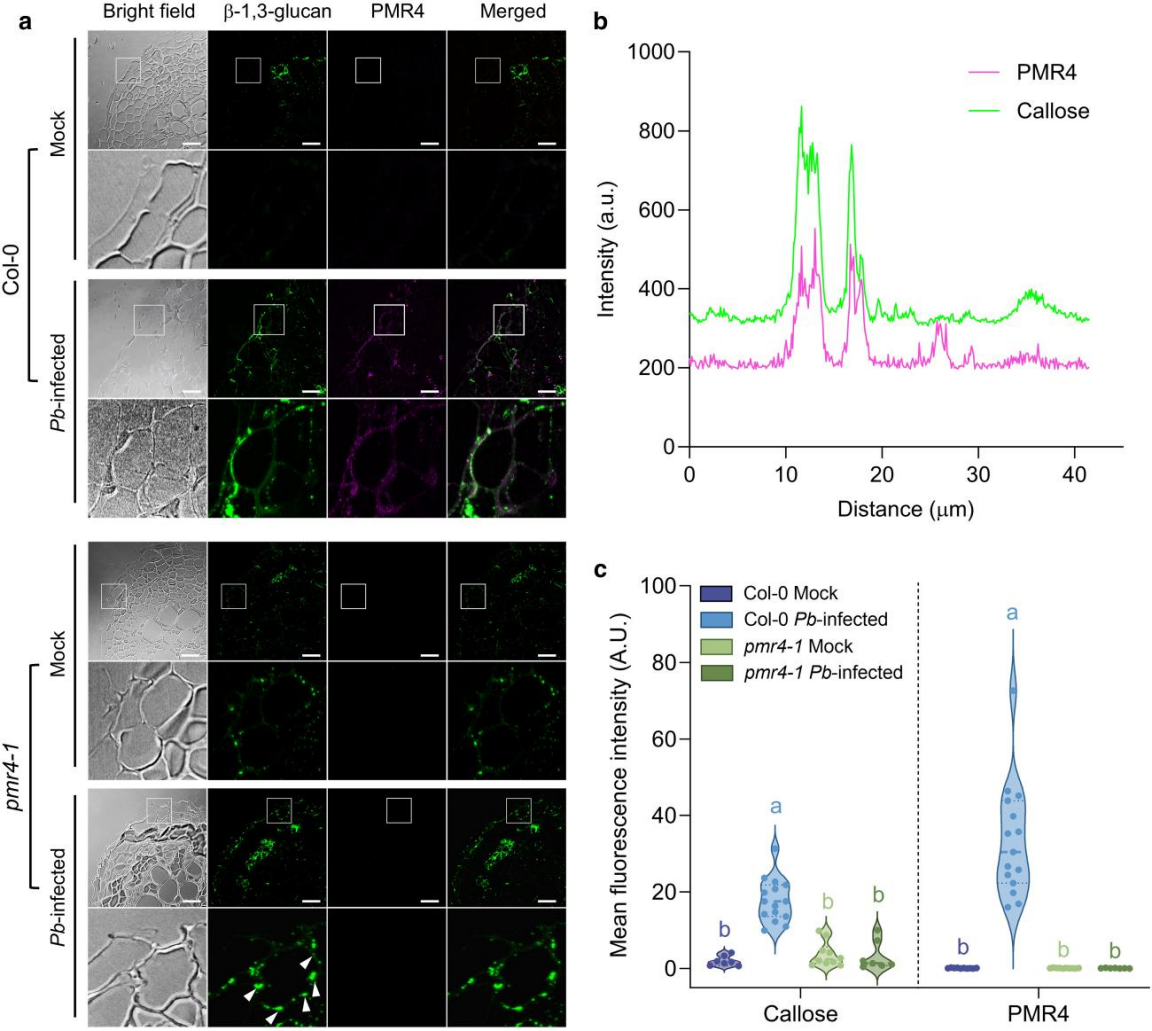
Immunohistochemical detection of callose and PMR4 in *Arabidopsis* roots. a) IHC results of callose and PMR4 protein from healthy and *Pb*-infected Col-0 and *pmr4-1* at 14 dpi. Callose and PMR4 antigens were detected by specific primary antibodies derived from mouse and rabbit hosts. Further visualization of 2 signals was performed by incubation of anti-mouse_Alexa_488-conjugated and anti-rabbit_Alexa_594-conjugated secondary antibodies. White arrowheads indicate the punctate PD-localized callose deposition. Regions containing periderm walls were marked by white squares on upper panels and magnified as shown in corresponding lower panels. Scale bar = 20 *µ*m. b) Signal correlation analysis of callose and PMR4 in *Pb*-infected Col-0 roots. A representative result from 10 measurements is shown. c) Statistical analysis of the mean fluorescence intensity observed from a). Ten to 15 randomly picked periderm fields from at least 5 independent roots were analyzed by Fiji software. Two-way ANOVA was used to show the statistical difference. (α = 0.05). Letters represent significant differences.

To further examine *Pb* infection-triggered callose deposition and PMR4 localization in *Arabidopsis* roots, we performed an immunohistochemistry (IHC) assay. Both mock and *Pb*-infected Col-0 and *pmr4-1* roots were incubated with β-1,3-glucan- and PMR4-specific primary antibodies, followed by corresponding fluorescent secondary antibodies to detect callose deposition and PMR4 distribution, respectively. In uninfected Col-0 and *pmr4-1* roots, only phloem callose signals were frequently observed (Fig. 6a), in agreement with the background callose detected in uninoculated *B. napus* roots stained with aniline blue (Fig. S13b). Since no PMR4 signal was detected overlaying with such phloem callose signals, the phloem callose synthesis is likely to be independent of PMR4. In contrast, a strong callose signal was observed from *Pb*-infected host cell wall apoplasts in Col-0 roots, which was proximal to the PMR4 signal found in plasma membrane, whereas no such callose and PMR4 distribution patterns could be observed from *Pb*-infected *pmr4-1* roots (Fig. 6a). Moreover, fluorescence signal correlation analysis of *Pb* infection-triggered callose and PMR4 suggests a spatial association between the 2 signals (Fig. 6b). Quantification of the mean fluorescence intensity in the periderm revealed elevated callose and PMR4 levels in *Pb*-infected Col-0 roots but not in *pmr4-1* roots (Fig. 6c), supporting the notion that *Pb* infection induces callose deposition in a PMR4-dependent manner. Interestingly, following *Pb* infection, a punctate callose deposition pattern was observed in aniline blue-stained *pmr4-3g37* roots (Fig. S13b, red arrowheads) and in IHC-visualized *pmr4-1* roots (Fig. 6a, white arrowheads). This pattern is characteristic of a plasmodesmata (PD)–associated callose deposition (Vatén et al. 2011) and reminiscent of the callose accumulation seen on *Ec*-infected *pmr4-1* leaves (Jacobs et al. 2003), suggesting possible activity of other callose synthase isoforms at PD, although the biological significance of this pattern remains unclear.

### Inactivation of JA pathway partially restores *pmr4-1* susceptibility to *Pb*

**Figure 7:**
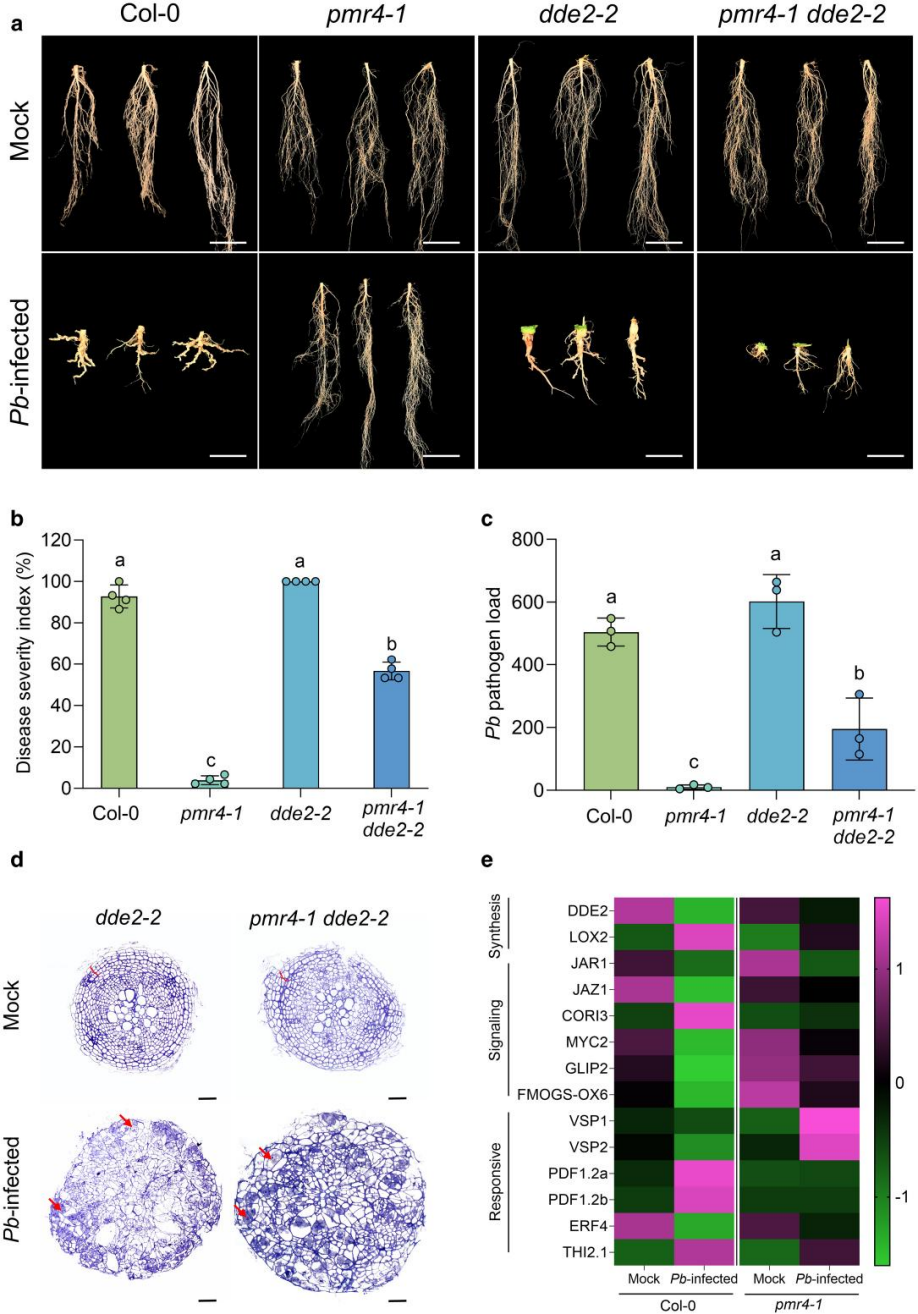
Effects of blocking JA signaling on *pmr4-1* root response to the *Pb* infection. a) Representative root images of Col-0, *pmr4-1*, and *pmr4-1*-associated mutants with and without *Pb* pathotype 3H inoculation at 21 dpi. Scale bar = 2 cm. b) Quantitative analysis of *Arabidopsis* clubroot disease severity as shown in a). DSI values are means ± Sd obtained from 3 groups of biological repeats, each containing 15 individual plants. One-way ANOVA was performed, and different letters represent various disease severity levels as well as statistical significance. c) Relative *Pb* biomass as measured by qPCR of a *Pb* internal transcribed spacer 1 region (Wen et al. 2020). *AtUBQ10* was used as a host tissue normalization reference. At least 3 plant roots were pooled to form 1 mixed sample with 3 biological replicates for each line. Data are means ± Sd obtained from 3 technical replicates. One-way ANOVA analysis for *Pb* relative biomass at 21 dpi was performed. Letters represent significant differences. d) Histological analysis of the roots of mock or *Pb*-inoculated *dde2-2* and *pmr4-1 dde2-2* plants at 14 dpi. Representative *Pb* secondary infections were indicated with arrowheads. Representative images were selected from the 5 independent roots with a similar phenotype. Red brackets indicate periderm. Scale bar = 1 mm. e) Transcriptome profiling of the gene expressions involved in JA synthesis, signaling, and response in the roots before or after *Pb* inoculation at 14 dpi. Heatmap was generated based on the *Z*-score from 3 biological repeats for each group of samples.

JA signaling is involved in wound- and pathogen-induced plant immunity (Koo and Howe 2009; Howe et al. 2018; Zhang et al. 2025) but not required for *pmr4*-mediated resistance to PM (Nishimura et al. 2003). In addition, JA plays a key role in mediating cell wall responses by regulating genes involved in cell wall remodeling and reinforcement during stress and developmental signaling (Howe et al. 2018). As PMR4-mediated callose deposition is a hallmark of cell wall reinforcement during pathogen invasion, we asked whether JA signaling is required for *pmr4*-conferred clubroot resistance by introducing the *dde2-2* mutation known to abolish JA biosynthesis (von Malek et al. 2002) into *pmr4-1* plants. Surprisingly, *Pb*-infected *pmr4-1 dde2-2* developed smaller root galls than Col-0 and *dde2-2* at 21 dpi, while swollen root tissues were rarely observed in *pmr4-1* roots (Fig. 7a). Quantitative analyses revealed a partial restoration of susceptibility with increased DSI (Fig. 7b) and pathogen load (Fig. 7c) in *pmr4-1 dde2-2* double mutants compared with *pmr4-1* single mutants. Furthermore, cytological examination of *Pb*-infected *pmr4-1 dde2-2* roots revealed massive secondary plasmodia (Fig. 7d) comparable to *Pb*-infected Col-0 roots (Fig. 5a). Together, these results indicate that *pmr4-1*-mediated resistance to clubroot requires intact JA signaling, similar to *Rcr1*-conferred clubroot resistance (Chu et al. 2014).

To further elucidate the molecular mechanisms underlying clubroot susceptibility and *pmr4-1*-mediated resistance, we performed RNA-seq analysis on Col-0 and *pmr4-1* plants following *Pb* inoculation (Fig. 7e). Transcriptomic profiling revealed that *Pb* infection suppressed the expression of several key genes involved in JA biosynthesis, signaling and response in Col-0 root tissues. In contrast, most of them were derepressed or even further induced (eg, *VSP1* and *VSP2*) in infected *pmr4-1* roots (Fig. 7e). Notably, many of these JA-related genes were also upregulated in noninoculated *pmr4-1* plants compared with Col-0, suggesting an antagonistic relationship between JA-mediated signaling and PMR4-dependent callose production. Transcriptomic changes in selected JA-related genes were further validated by RT-qPCR, yielding results consistent with the RNA-seq analysis (Fig. S14).

Collectively, these results demonstrate that *Pb* infection suppresses JA signaling in Col-0 roots, whereas loss of PMR4 function leads to derepressing JA signaling in *pmr4-1* roots. The elevated JA pathway activity in *pmr4-1* plants likely contributes to their enhanced resistance to clubroot.

### JA signaling is required for and positively correlates to lignin-like compound synthesis and possibly suberin deposition in the periderm

**Figure 8:**
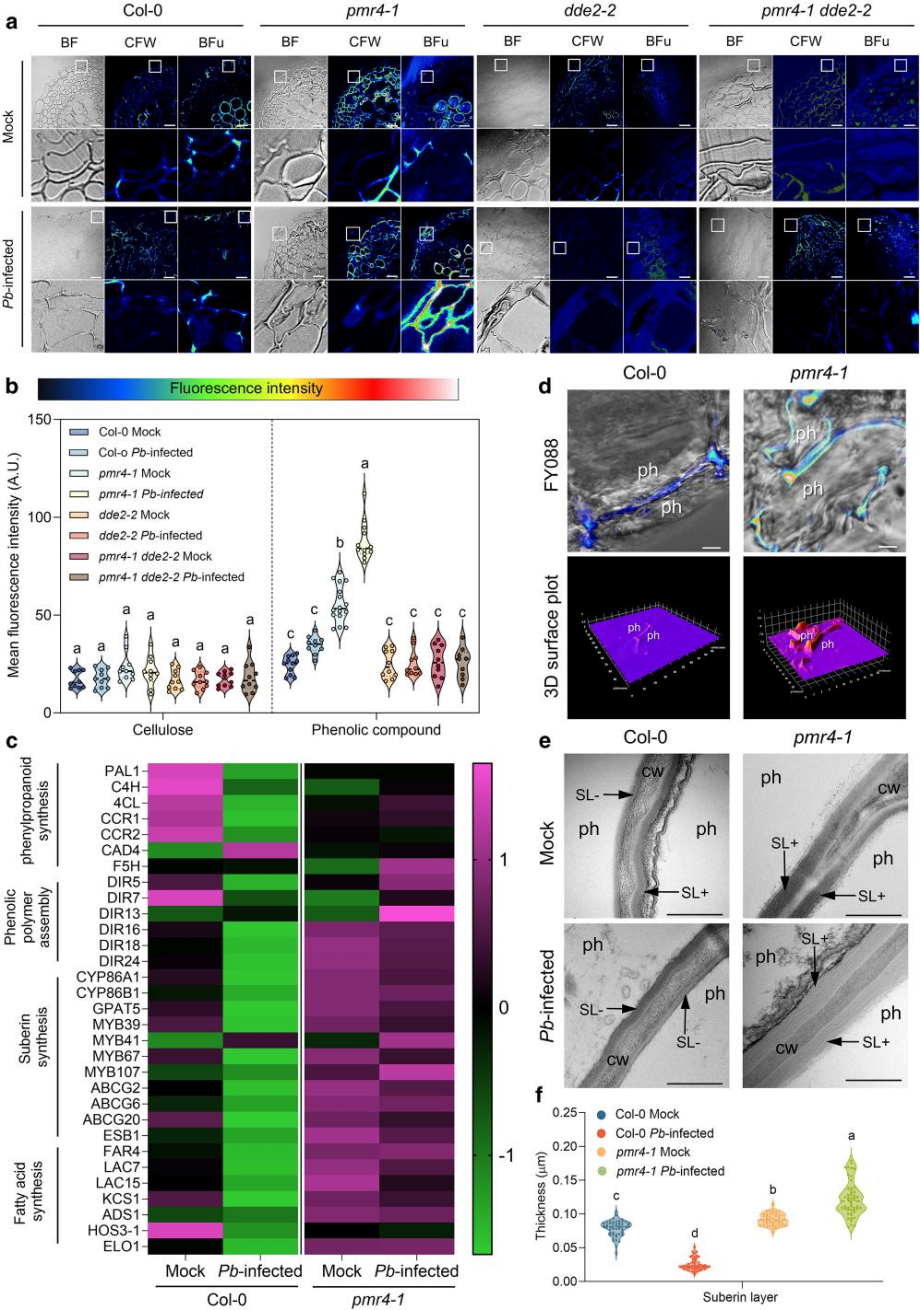
Effects of *pmr4-1* mutation and JA pathway on periderm root cell wall composition. a) CLSM-based cellulose and lignin-like phenolic compound detection in Col-0, *pmr4-1*, and JA-deficient-associated mutants. Cellulose and lignin-like compounds were stained with CFW and BFu, respectively. White squares marking periderm were enlarged below each corresponding image. Scale bars = 20 *µ*m. A pseudocolor scheme was given to visualize the spatial distribution and relative abundance of the detected cell wall components, with colors ranging from dark to bright indicating increasing fluorescence intensity. BF, bright field. b) Statistics of the relative deposition amount of cellulose and lignin-like phenolic compound observed from a). The periderm was selected for mean fluorescence intensity analysis by Fiji software. Ten randomly selected fields from 5 independent roots were calculated, and ordinary 1-way ANOVA was used to show the statistical significance (α = 0.05). Letters represent significant differences. c) Heatmap of transcriptome profiling of the gene expressions involved in phenylpropanoid synthesis, phenolic polymer assembly, suberin synthesis, and fatty acid synthesis processes in the roots with or without *Pb* inoculation at 14 dpi from Col-0 and *pmr4-1*. Heatmap was generated based on the *Z*-score from 3 biological repeats for each group of samples. d) Suberin detection from *Pb*-infected Col-0 and *pmr4-1* at 14 dpi. Cryostat-sections of *Pb*-infected Col-0 and *pmr4-1* roots at 14 dpi were stained with FY088 and observed under CLSM. Representative images focusing on the periderm phellem cell wall (upper row) were shown along with corresponding 3D surface plots (lower row). Scale bars = 100 *µ*m. e) TEM images of periderm phellem cell wall. CW, polysaccharide cell wall; SL+, suberin layer with a lamellated substructure; SL−, a largely amorphous suberin layer with little substructure; ph, phellem. Scale bars = 0.5 *µ*m. f) Suberin layer thickness. The thickness of the suberin layer visualized in TEM was measured in at least 4 phellem cells by measuring the thickness of the suberin lamellae. Values represent the means ± Sd, *n* ≥ 50. Different lowercase letters indicate significant differences, as determined by 1-way ANOVA, α = 0.05.

Thickened periderm cell walls in *pmr4-1* roots, as observed by TEM (Figs. 5d and  S12b), suggest that the altered cell wall architecture plays a role in the clubroot resistance. We investigated potential cellular and molecular mechanisms underlying this phenotype using several approaches. Firstly, UDP-Glc serves as a precursor for both callose and cellulose biosynthesis; hence, the loss of PMR4 callose synthase might tip the balance toward cellulose accumulation in cell walls (Liu et al. 2023). Surprisingly, no significant difference in cellulose abundance, as revealed by calcofluor white (CFW) fluorescence (Ursache et al. 2018), was observed among tested lines regardless of the *Pb* infection (Fig. 8a and b), indicating that the cellulose content in *pmr4-1* cell walls is unaltered and does not contribute to *pmr4*-mediated clubroot resistance.

Secondly, wounding triggers JA signaling and promotes lignin deposition around wound sites in plant tissues (Xu et al. 2024). Since JA signaling is required for *pmr4*-mediated clubroot resistance (Fig. 7), we also assessed the deposition of lignin-like phenolic compounds using a histochemical approach. Basic fuchsin (BFu) staining, which specifically labels lignified cell walls (Ursache et al. 2018), revealed increased lignin-like phenolic components in noninfected *pmr4-1* roots compared with Col-0, with a further increase following *Pb* infection. In contrast, BFu fluorescence was barely detectable in JA-deficient *dde2-2* mutants, regardless of *PMR4* genotype or infection status (Fig. 8a and b). As a complementary approach, cell wall autofluorescence detected using 405-nm laser-based confocal microscopy (Perez-de-Lis et al. 2024) showed consistent patterns of lignin-like phenolic polymer deposition (Fig. S15a and b).

Thirdly, RNA-seq transcriptomic analysis revealed that genes associated with phenylpropanoid biosynthesis, as well as the assembly and deposition of phenolic polymers, were significantly suppressed in Col-0 roots following *Pb* infection, whereas these genes remained active or were even induced in *pmr4-1* roots. Lignification in developing *Arabidopsis* root tissues is closely linked to the suberin layer formation at the inner cell walls of periderm cells, which involves cross-linking between aromatic phenolic compounds and fatty alcohols (Wunderling et al. 2018; Gully et al. 2024). Consistent with this relationship, genes involved in fatty acid biosynthesis, which provide specific precursors for suberin production, exhibited expression patterns similar to those of phenolic compound biosynthesis and polymerization genes. Similarly, genes associated with suberin biosynthesis and deposition showed comparable expression trends (Fig. 8c), and these patterns were further validated by RT-qPCR analysis (Fig. S16). Collectively, genes involved in phenylpropanoid biosynthesis and polymerization, fatty acid biosynthesis, and suberin formation and deposition were coordinately regulated during clubroot infection and in the *pmr4-1* mutant. These results suggest that suberization is downregulated in root tissues during susceptible clubroot disease development but is upregulated upon loss of PMR4 function.

To investigate suberization in root tissues during *Pb* infection, we utilized fluorol yellow 088 (FY088) staining to visualize suberin deposition (Chen et al. 2024). Following infection, strong FY088 fluorescence was observed at the phellem cell walls of *pmr4-1* roots, whereas weaker signals were detected in Col-0 (Fig. 8d, upper row). A 3D surface plot further confirmed enhanced suberin accumulation in *pmr4-1* compared with Col-0 (Fig. 8d, lower row). TEM imaging (Fig. 8e) of the same root regions revealed distinct suberin lamella structures between Col-0 and *pmr4-1*. In Col-0 roots, cell walls displayed high electron density with fewer lamella layers, indicative of predominantly aliphatic-rich suberin. In contrast, *Pb*-infected *pmr4-1* roots exhibited multiple layers of a lamellated, low electron density suberin, consistent with an aromatic-enriched structure (Gully et al. 2024). Notably, while *Pb* infection compromised the suberin layers in Col-0 roots, *pmr4-1* roots showed substantial suberin thickening (Fig. 8f). The periderm tissues of *pmr4-1*, enriched in phenolic polymers and suberin, resemble the cork layer found in woody plants (Teixeira 2022) and could serve as a physical barrier restricting pathogen spread within root tissues during secondary infection.

## Discussion

The Brassicaceae family includes many valuable crops, among which canola varieties of *B. napus* are cultivated worldwide for edible oil and other applications due to their high nutritional value and energy outputs. Clubroot disease poses a major threat to oilseed production worldwide. Growing *CR* cultivars remains the most effective disease management strategy; however, resistance conferred by *CR* genes can be overcome readily due to the pathogen diversity and evolution. *CR* genes have been found mainly in other *Brassica* species that often need to be introduced into *B. napus* varieties through traditional breeding, which is time consuming and difficult to keep pace with the evolving pathogen population (Hwang et al. 2012). Recently, a novel gene *WeiTsing* (*WTS*) from *Arabidopsis* was identified and characterized. *B. napus* plants carrying *WTS* display a broad-spectrum clubroot resistance (Wang et al. 2023), making it a promising *CR* gene for clubroot management. In this study, we developed a strategy to screen available *Arabidopsis* mutants aiming at identifying candidate *CS* genes that can be edited in Brassicaceae crops with potential durable clubroot resistance against multiple *Pb* pathotypes.

Our genetic screen to date has identified several *Arabidopsis* mutant lines displaying various levels of clubroot resistance, and one of them (*pmr4-1*) showed strong resistance. Several pieces of evidence in this study support the conclusion that *PMR4*, a previously reported *S* gene for PM (Nishimura et al. 2003), is also an *S* gene for clubroot. Firstly, 2 mutant lines carrying independent alleles, *pmr4-1*, containing a premature stop codon in Exon 2, and *gsl5-1*, a T-DNA insertion in the same exon, showed strong resistance to clubroot. Secondly, although it is unclear whether *pmr4-1* and *gsl5-1* are null mutations, they behave as loss-of-function alleles and are indeed recessive mutations. Thirdly, in *B. napus*, resistance was observed only when both *PMR4* orthologs were disrupted by gene editing, indicating functional redundancy. Finally, wound- and pathogen-induced callose deposition experiments confirmed that both *Arabidopsis* and *B. napus pmr4* mutants are loss-of-function mutations. Although other *CS* genes have been previously reported (Chen et al. 2016; Walerowski et al. 2018), *PMR4*, to the best of our knowledge, is the first *CS* gene whose inactivation results in such strong clubroot resistance. Since the *PMR4* gene is highly conserved within the Brassicaceae family, we created the corresponding *pmr4* double mutant lines in *B. napus* by genome editing and demonstrated that they were dually resistant to both clubroot and PM diseases. Since the PMR4 callose synthase activity is stress-induced (Jacobs et al. 2003; Nishimura et al. 2003) and *pmr4-1* mutant plants do not exhibit apparent penalties under normal growth conditions, we anticipate that *PMR4* orthologs in Brassicaceae crops can be targeted via gene editing to enhance clubroot resistance while maintaining desired agronomic traits, although field-level fitness remains to be confirmed.

Perhaps the most striking findings from this study were that, while the PM resistance conferred by *pmr4-1* depended on the functional SA but not JA pathway (Nishimura et al. 2003), its resistance to the clubroot disease was dependent on JA but independent of SA response, suggesting that *pmr4*-*1* confers resistance to these 2 pathogens through distinct mechanisms. In the case of PM resistance, the elevated SA signaling in *pmr4* mutants was regarded as an underlying mechanism (Nishimura et al. 2003). Our studies convincingly demonstrated that, unlike observations in leaves, the *pmr4-*mediated clubroot resistance was largely due to derepressed JA response in roots. Our experimental data favor a notion that PMR4 functions as a host susceptibility factor required for the completion of *Pb* infection cycle. Firstly, *Pb* induced PMR4-dependent callose deposition in *Arabidopsis* and *B. napus* roots, reminiscent of wound- and *Ec*-induced callose deposition in *Arabidopsis* leaves (Jacobs et al. 2003). Secondly, we showed that *pmr4-1* blocks *Pb* secondary infection, resulting in an incomplete *Pb* disease cycle. Notably, primary infection often proceeds in *CR* canola varieties (Xu et al. 2018; Wang et al. 2023; Tu et al. 2024) and even in some nonhosts (Liu et al. 2020b), reinforcing the notion that restricting *Pb* secondary infection is the primary target to achieve clubroot resistance. Finally, quantitative pathogen biomass analysis confirms that disrupting PMR4 callose synthase suppresses *Pb* proliferation and gall formation.

**Figure 9:**
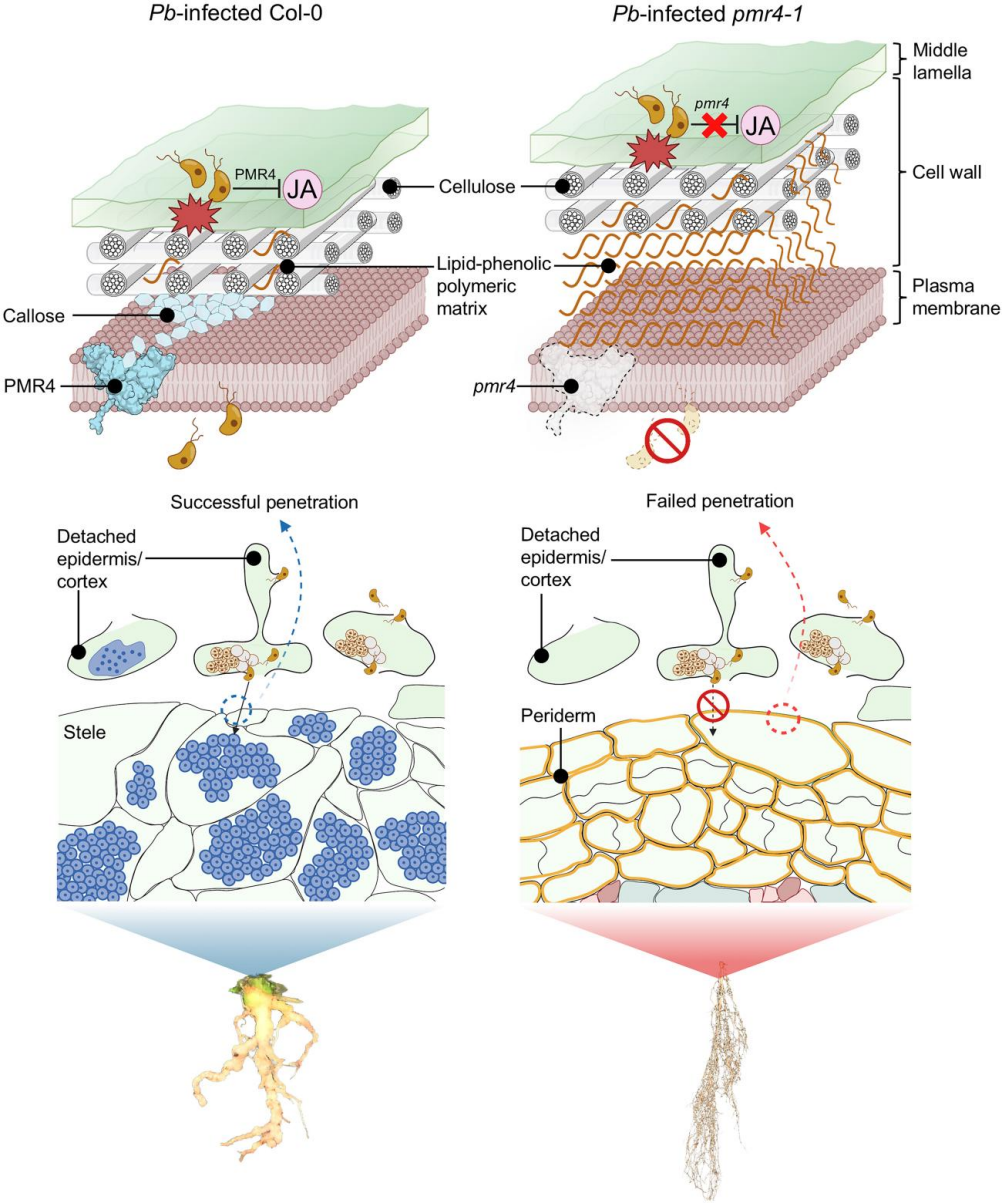
A working model depicting mechanisms of *pmr4-*mediated resistance to *Pb* in roots. *Pb* zoospores are released from zoosporangium in root hairs or epidermal cells in both Col-0 and *pmr4-1*, indicating successful primary infection. However, while *Pb* pathogen can complete its disease cycle in Col-0 roots and form resting spores in cortical cells and the stele, secondary infection is rarely found in *pmr4-1* roots. Consequently, *Pb* infection causes giant galls in Col-0 roots, while *pmr4-1* roots barely develop symptoms. Diagrams on top depict the host periderm cell wall or equivalent layers enlarged from dashed circles. In Col-0 roots, *Pb* invasion triggers the PMR4-dependent callose deposition. JA signaling is repressed upon pathogen infection, leading to compromised lignin-like phenolic compound and suberin syntheses. In *pmr4-1* periderm, lack of PMR4-dependent callose deposition correlates with derepressed JA signaling and enhanced synthesis of lignin-like phenolic compounds and suberin, which cross-link other cell wall components to form a physical barrier to prevent *Pb* zoospore penetration into the stele.

This study suggests that *pmr4*-mediated and JA signaling–dependent periderm cell wall reinforcement plays a critical role in the protection against *Pb* infection. Firstly, TEM image analysis revealed thickened root periderm cell walls in *pmr4-1* and particularly in response to the *Pb* infection; in contrast, the mature periderm was not observed in *Pb*-infected Col-0 roots, suggesting that *Pb* infection is associated with incomplete periderm maturation in roots of a susceptible host. Secondly, lignin-like and suberin compounds were accumulated more in *pmr4-1* than in Col-0 cell walls and likely formed specialized structures. Thirdly, the above changes were positively correlated with transcriptomic data showing increased transcript levels in related biosynthetic pathways. Finally, the increased lignin-like compounds in *pmr4-1* roots depended on functional JA signaling, which was also correlated with the JA pathway transcriptomic profile. Based on the above analyses, we propose a working model as shown in Fig. 9, in which the *Pb* pathogen suppresses JA signaling and prevents periderm maturation to facilitate its secondary infection. Loss of PMR4 reinstates JA signaling, leading to increased phenolic compound and suberin synthesis to promote root periderm maturation, which in turn forms a barrier to prevent *Pb* secondary infection. Apparently, several questions remain to be addressed by future investigation. For example, is enhanced JA signaling alone sufficient to promote phenolic compound biosynthesis and root periderm maturation? Is lignin-like or suberin, or both in periderm, required for the protection against *Pb* infection? How does the *Pb* pathogen utilize PMR4 callose synthase to inhibit JA signaling?

While this study was in progress (initial manuscript was submitted and posted on bioRxiv on 19 September 2024), Dr Liu and his colleagues reported similar findings (Wu et al. 2025). Hence, 2 research teams independently found that loss of PMR4/GSL5 confers strong clubroot resistance in a JA-dependent but SA-independent manner. Our studies report that JA-dependent root periderm cell wall homeostasis plays a critical role in *pmr4*-mediated clubroot resistance. Since the JA signaling plays important roles in secondary cell wall development by promoting lignin and suberin biosynthesis (Hyde et al. 2018; Im et al. 2024; Xu et al. 2024; Jiang et al. 2025), which are characteristic compounds found in periderm tissues (Wunderling et al. 2018; Serra et al. 2022) and have been implicated in defense against both biotic (Du et al. 2011; Thangavel et al. 2016) and abiotic (Lux et al. 2011; Ranathunge et al. 2016; de Silva et al. 2021) stresses, the *pmr4* mutation may constitute even broader stress responses in higher plants.

## Materials and methods

### Plant materials and growth conditions

*A. thaliana* wild-type (Columbia-0) and mutant lines used in this study were either purchased from ABRC (https://abrc.osu.edu/) and NASC (https://arabidopsis.info/BasicForm) or kindly provided by other research groups. Detailed information is listed in Table S1. All oligonucleotides used as primers for genotyping, real-time PCR and ddPCR analyses are listed in Table S2. A doubled haploid canola (*B. napus*) cultivar DH12075 was used to generate transgenic *pmr4-3g37* and *pmr4-3g41* plants as described below. For *Arabidopsis* seedling culture, seeds were sterilized in 10% bleach for 15 min, rinsed in sterile ddH_2_O 3 times, and then germinated on a 1/2 strength MS medium containing 0.6% agar. For clubroot or PM disease tests, *Arabidopsis* and *B. napus* seeds were sown and grown in the soil Sunshine Mix #5 Natural & Organic (Sun Gro Horticulture Canada Ltd). *Arabidopsis* and *B. napus* plants were grown at 22 °C under 16 h ∼125 *μ*E m^−2^ s^−1^ light and 8 h dark photoperiod conditions.

### *Pb* and *Ec* inoculation

Resting spore inocula from *Pb* pathotypes 3H, 3A, 5X-LG2, and 5X-LG3, the designation based on Canadian Clubroot Differential system (Tso et al. 2021), were prepared using galled roots of infected *B. napus* plants. The galled roots were homogenized in a blender with distilled water, and then plant residues were removed from the suspension by filtering through 8 layers of cheesecloth. The resting spore density was measured by hemocytometry and then diluted in ddH_2_O to a final density of 1 × 10^7^ and 1 × 10^8^ spores per mL for *Arabidopsis* and *B. napus* inoculation, respectively. For the *Arabidopsis* inoculation, each 14-d-old seedling was inoculated with 1 mL of resting spore inoculum. *B. napus* were inoculated twice with 1 mL spore inoculum each, once at the seeding and a second time at 7 d after planting when seedlings emerged. For both *Arabidopsis* and *B. napus* plants, the clubroot disease severity was rated using a modified 0 to 3 scoring system (Kuginuki et al. 1999). A score of 0 indicates no disease; 1, very small galls on the lateral roots but without deformation on the primary roots; 2, medium size galls on primary roots; and 3, severe giant gall formations in the primary roots with or without very few swollen lateral roots left. The clubroot symptoms were rated for 3 to 4 biological replicates, and every 15 plants were pooled and rated as 1 biological repeat. The DSI was calculated according to the formula:


$$\mathrm{DSI}=\frac{\sum \left(\mathrm{rating}\;\mathrm{class})\times (\mathrm{number}\;\mathrm{of}\;\mathrm{plants}\;\mathrm{in}\;\mathrm{the}\;\mathrm{rating}\;\mathrm{class}\right)}{\mathrm{total}\;\mathrm{number}\;\mathrm{of}\;\mathrm{plants}\;\mathrm{times}3}\times 100$$

*Ec* was maintained and propagated on *B. napus* plants. Approximately 4-wk-old *Arabidopsis* plants were inoculated with *Ec* conidiospores at a density of 5 to 10 conidia/mm^2^. For *B. napus*, tested plants were placed in settling towels and inoculated with conidia by tapping the infected *B. napus* leaves above the tested plants. The inoculated plants were kept away from air flows for at least 3 h after the conidia inoculation to obtain the desired germination rates of the pathogen.

### ddPCR and RT-qPCR

For the sampling of *Pb-*inoculated root samples, 5 root galls or 6 to 7 nonswollen roots were pooled as 1 biological repeat, with at least 3 repeats for each treatment. Root samples were collected and cut by scissors and ground by Lysing Matrix Tubes (FastPrep) or by liquid nitrogen. The homogenized mixture was used either for genomic DNA extraction by using a DNeasy Plant Mini Kit (QIAGEN) or for total RNA extraction using a RNeasy Plant Mini kit (QIAGEN) following the instructions.

The biomass of *Pb* was quantified by qPCR or ddPCR using the QX200 ddPCR System (Bio-Rad) following the manufacturer's instructions. For each ddPCR sample, a 20-*µ*L reaction contained 10 *µ*L of probe supermix (Bio-Rad), 1 *µ*L each of 22.5 *µ*m forward and reverse primers, 1 *µ*L of 6.25 *µ*m probe, and 2 *µ*L genomic DNA. The mixture was partitioned into droplets using a droplet generator, transferred to a 96-well PCR plate and followed by a standard PCR reaction. Droplets were read using the droplet reader, and data were analyzed with QuantaSoft Software (Bio-Rad). A fluorescence threshold of 2,000 was applied to distinguish positive droplets from background signals.

For RT-qPCR, the first-strand cDNA was synthesized from RNA using an iScript cDNA Synthesis kit (Bio-Rad). qPCR was performed against the first-strand cDNA in a qPCR detection system using the iQ SYBR Green Supermix and a passive reference dye (Bio-Rad). The qPCR was run in a 20 *μ*L mixture containing 0.5 *μ*L each of 10 *μ*m forward and reverse primers and 100 ng of the cDNA template. The calculation for each sample was based on the average of 3 technical replicates. The ΔΔCT method (Livak and Schmittgen 2001) was used to analyze relative transcript abundance. Gene expression was normalized to the endogenous housekeeping genes *AtUBQ10* or *PbActin1* as indicated in the corresponding figure legends.

### Histochemical staining of plant samples

To observe PM structures, *Ec*-inoculated leaves were detached and collected at different time points and fixed in 1:3 (v:v) acetic acid:ethanol. An acidic aniline blue solution (2 mg/mL, pH 5.0) was used to stain *Ec* fungal structures.

*Pb* primary infection and PM-triggered callose deposition were monitored as previously described by Liu et al. (2020a) and Qin et al. (2021), respectively.

To examine root morphology by Toluidine Blue-O, the entire procedure from sample collection to microscope observation was performed as previously described (Tu et al. 2024).

To detect callose deposition in *Pb-*infected *B. napus* roots, lateral root samples proximal to hypocotyl were harvested at 14 dpi and fixed overnight with a solution containing 37% formaldehyde, glacial acetic acid, 95% ethanol and deionized water at a volume ratio of 50:5:10:35. Samples were then embedded in the Tissue Plus O.C.T compound (Leica Microsystems), sectioned with a Leica CM1860 cryostat microtome to 50 *µ*m, stained with 0.05% to 0.1% aniline blue solution for 15 min and rinsed 3× 5 min with distilled water before analysis.

To observe cellulose and lignin-like compounds, Electron Microscopy Sciences LR White-embedded roots were cross-sectioned into 0.5-*μ*m thick by an ultramicrotome (Reichert-Jung Ultracut E Ultramicrotome) before being collected on poly-L-lysine-coated slides. 0.1% (w/v) CFW and 0.1% (w/v) BFu dissolved in ClearSee solution were applied onto the root cross-section for overnight staining at 4 °C. Slides were rinsed 3× 5 min with distilled water before mounting.

To observe suberin in *Arabidopsis* cryostat cross-sections, a 0.4% (w/v) FY088 stock solution in DMSO was prepared. 0.01% (v/v) FY088 diluted in lactic acid was applied to incubate the root samples at 70 °C for 30 min, followed by 2× 5 min rinse with lactic acid at 70 °C before mounting slides.

### Microscopy observations

Light microscopic images were taken using a Zeiss Axioplan 2 upright fluorescence microscope. Confocal images were taken on a Zeiss LSM 880 confocal laser scanning microscope. Excitation/emission wavelengths were 405/445 to 480 nm for DAPI and CFW, 405/480 to 515 nm for aniline blue, 405/425 to 523 nm for autofluorescence, 488/499 to 540 nm for HCS LipidTox Green and GFP, 488/490 to 550 nm for FY088, 543/600 to 650 nm for BFu, and 594/599 to 734 nm for RFP.

### Immunofluorescence

Both healthy and *Pb*-infected upper lateral roots ∼1 cm from the hypocotyl were collected, and specimens were prepared as described (Amsbury and Benitez-Alfonso 2022). Vector TrueVIEW autofluorescence quenching kit (MJS BioLynx, VECTSP8400) was used following the instructions to reduce autofluorescence. The specimens were mounted with VECTASHIELD Vibrance Antifade mounting media (Vector Laboratories, H-1700) and examined with Zeiss 880 confocal microscope. Antibodies used in this study include β-1,3-glucan-specific (Biosupplies 400-2, 1:80 dilution) and PMR4-specific (Agrisera AS21 4567, 1:50 dilution) primary antibodies, anti-mouse_Alexa_488 (R&D System IC002G, 1:100 dilution) and anti-rabbit_Alexa_594 (Invitrogen A-21207, 1:100 dilution) secondary antibodies.

### Transmission electron microscopy

Polysciences Spurr Low Viscosity Embedding Kit (01916-1) was used to embed root samples. Ultra-thin cross-sections of around 90 nm were collected onto formvar-coated 1 × 2 mm slotted Cu grids and stained with 2% uranyl acetate for 12 min by floating on a drop of stain. After an extensive wash with distilled water, the specimen was stained with Reynold's Lead citrate solution (Ted Pella, Inc.) for 6 min in a closed chamber, dipped in 0.02 N NaOH 20 times, washed in distilled water, and then observed with a Hitachi HT7700 transmission electron microscope.

### Cell wall component measurements

Fiji software (Schindelin et al. 2012) was used for analysis. For the measurement of mean fluorescence intensity in a given field, all analyzed CLSM-based images were collected from the same round of assay under the same treatment conditions. The same parameter and threshold were applied. For the measurement of cell wall area or thickness, all analyses were based on the TEM images. TEM images were first fed into Fiji software. The cell area was measured by selecting along the plasma membrane, while cell wall thickness was determined by the distance between adjacent cell plasma membranes.

### CRISPR/Cas9 vector assembly

Sequence-specific single-guide RNAs (sgRNAs) were designed by using a web-based tool CRISPR-P (http://crispr.hzau.edu.cn/cgi-bin/CRISPR2/CRISPR). Target sites were selected based on their location in the target genes, GC% content and putative off-targets. sgRNA-F/R primers (Table S2) were annealed and inserted between 2 *Bsa*I sites of a binary vector pHEE401 obtained from Dr Qijun Chen (China Agriculture University, China), resulting in pHEE-BnaPMR4-sgRNAs. Plasmid pHEE401 contains a hygromycin resistance marker driven by a cauliflower mosaic virus *35S* promoter, a Cas9 coding sequence driven by an egg cell–specific promoter *EC1.2*, and the cloned sgRNA is driven by a *U6-26p* promoter (Wang et al. 2015).

### *B. napus* transformation

The *B. napus* doubled haploid line DH12075 was used as the transformation recipient in this study. The *Agrobacterium tumefaciens*–mediated hypocotyl transformation protocol (Zhou et al. 2002) was followed.

### Transgenic plant genotyping

Genomic DNA from leaf tissues were used as PCR templates to amplify DNA flanking the CRISPR target sites using specific primers (Table S2). The PCR products were directly sequenced, and the sequencing chromatograms were analyzed to identify and distinguish mutated from wild-type sequences adjacent to the protospacer adjacent motif (PAM).

### Transcriptome profiling

The roots of *Arabidopsis* plants with or without *Pb* inoculation were harvested at 14 dpi with 3 replicates, in which 5 to 10 individual plants were pooled, and immediately frozen in liquid nitrogen. High-quality RNA was extracted using the RNeasy Kit (QIAGEN #74104). cDNA libraries were prepared using the Illumina TruSeq V1.5 Kit. Paired-end sequencing was performed at the Next-Generation Sequencing Facility, University of Saskatchewan, using NextSeq 2000 sequencing platform. Raw reads prefiltering was performed using Trimmomatic (Bolger et al. 2014) to trim the adaptor sequences, filter low-quality reads and eliminate short reads. STAR (Dobin et al. 2013) was used for fast and accurate alignment of cleaned reads to the *A. thaliana* TAIR10 reference genome. Reads quantification was performed using the Salmon tool (Patro et al. 2017). To summarize expression levels from the transcript level to the gene level, we used the R tximport package (Soneson et al. 2015) to process the outputs from Salmon. Expression matrixes between genes and all samples were then generated, and differentially expressed gene analysis was performed through R DESeq2 package (Love et al. 2014). Heatmaps were generated based on *Z*-score, which is calculated as log_2_ (TPM + 1) (transcript per million reads [TPM]). The RNA-seq data were submitted to the Gene Expression Omnibus repository (accession number GSE317839).

## Supplementary Material

koag160_Supplementary_Data

## Data Availability

The RNA-seq data were submitted to the Gene Expression Omnibus repository (accession number GSE317839).

## References

[koag160-B1] Amsbury S, Benitez-Alfonso Y. 2022. Immunofluorescence detection of callose in plant tissue sections. Methods Mol Biol. 2457:167–176. 10.1007/978-1-0716-2132-5_10. 35349139

[koag160-B2] Bolger AM, Lohse M, Usadel B. 2014. Trimmomatic: a flexible trimmer for Illumina sequence data. Bioinformatics. 30:2114–2120. 10.1093/bioinformatics/btu170. 24695404 PMC4103590

[koag160-B3] Boubakri H . 2023. Recent progress in CRISPR/Cas9-based genome editing for enhancing plant disease resistance. Gene. 866:147334. 10.1016/j.gene.2023.147334. 36871676

[koag160-B4] Bulman S et al 2019. *Arabidopsis thaliana* expressing PbBSMT, a gene encoding a SABATH-type methyltransferase from the plant pathogenic protist *Plasmodiophora brassicae*, show leaf chlorosis and altered host susceptibility. Plant Biol (Stuttg). 21:120–130. 10.1111/plb.12728. 29607585

[koag160-B5] Cardoza V, Stewart CN. 2006. Canola (*Brassica napus* L.). In: Wang K, editor. Methods in molecular biology, Vol. 343. Humana Press. p. 257–266. 10.1385/1-59745-130-4:257. 16988350

[koag160-B6] Chen T et al 2016. *Arabidopsis* mutant bik1 exhibits strong resistance to *Plasmodiophora brassicae*. Front Physiol. 7:402. 10.3389/fphys.2016.00402. 27679580 PMC5020103

[koag160-B7] Chen X et al 2024. Four MYB transcription factors regulate suberization and nonlocalized lignification at the root endodermis in rice. Plant Cell. 37:koae278. 10.1093/plcell/koae278. 39405464 PMC11663582

[koag160-B8] Chu M et al 2014. Fine mapping of Rcr1 and analyses of its effect on transcriptome patterns during infection by *Plasmodiophora brassicae*. BMC Genomics. 15:1166. 10.1186/1471-2164-15-1166. 25532522 PMC4326500

[koag160-B9] Consonni C et al 2006. Conserved requirement for a plant host cell protein in powdery mildew pathogenesis. Nat Genet. 38:716–720. 10.1038/ng1806. 16732289

[koag160-B10] de Silva NDG et al 2021. Root suberin plays important roles in reducing water loss and sodium uptake in *Arabidopsis thaliana*. Metabolites. 11:735. 10.3390/metabo11110735. 34822393 PMC8618449

[koag160-B11] Delaney TP et al 1994. A central role of salicylic acid in plant disease resistance. Science. 266:1247–1250. 10.1126/science.266.5188.1247. 17810266

[koag160-B12] Djavaheri M et al 2019. Mimicking the host regulation of salicylic acid: a virulence strategy by the clubroot pathogen *Plasmodiophora brassicae*. Mol Plant Microbe Interact. 32:296–305. 10.1094/MPMI-07-18-0192-R. 30199341

[koag160-B13] Dobin A et al 2013. STAR: ultrafast universal RNA-seq aligner. Bioinformatics. 29:15–21. 10.1093/bioinformatics/bts635. 23104886 PMC3530905

[koag160-B14] Doudna JA, Charpentier E. 2014. Genome editing. The new frontier of genome engineering with CRISPR-Cas9. Science. 346:1258096. 10.1126/science.1258096. 25430774

[koag160-B15] Du Y, Zhao L, Su Y. 2011. Tantalum (oxy)nitrides: preparation, characterisation and enhancement of photo-Fenton-like degradation of atrazine under visible light. J Hazard Mater. 195:291–297. 10.1016/j.jhazmat.2011.08.042. 21885191

[koag160-B16] Gao C . 2021. Genome engineering for crop improvement and future agriculture. Cell. 184:1621–1635. 10.1016/j.cell.2021.01.005. 33581057

[koag160-B17] Gao Q et al 2022. A receptor-channel trio conducts Ca^2+^ signalling for pollen tube reception. Nature. 607:534–539. 10.1038/s41586-022-04923-7. 35794475 PMC9308748

[koag160-B18] Gully K et al 2024. The GPAT4/6/8 clade functions in *Arabidopsis* root suberization nonredundantly with the GPAT5/7 clade required for suberin lamellae. Proc Natl Acad Sci U S A. 121:e2314570121. 10.1073/pnas.2314570121. 38739804 PMC11127019

[koag160-B19] Hofmann RM, Pickart CM. 1999. Noncanonical MMS2-encoded ubiquitin-conjugating enzyme functions in assembly of novel polyubiquitin chains for DNA repair. Cell. 96:645–653. 10.1016/S0092-8674(00)80575-9. 10089880

[koag160-B20] Hollman KB et al 2023. Characterization of pathotypes from western Canada in 2019–2020. Can J Plant Pathol. 45:475–484. 10.1080/07060661.2023.2212639.

[koag160-B21] Howard RJ, Strelkov SE, Harding MW. 2010. Clubroot of cruciferous crops - new perspectives on an old disease. Can J Plant Pathol. 32:43–57. 10.1080/07060661003621761.

[koag160-B22] Howe GA, Major IT, Koo AJ. 2018. Modularity in jasmonate signaling for multistress resilience. Annu Rev Plant Biol. 69:387–415. 10.1146/annurev-arplant-042817-040047. 29539269

[koag160-B23] Hsieh YSY, Kao MR, Tucker MR. 2024. The knowns and unknowns of callose biosynthesis in terrestrial plants. Carbohydr Res. 538:109103. 10.1016/j.carres.2024.109103. 38555659

[koag160-B24] Hwang SF, Strelkov SE, Feng J, Gossen BD, Howard RJ. 2012. *Plasmodiophora brassicae*: a review of an emerging pathogen of the Canadian canola (*Brassica napus*) crop. Mol Plant Pathol. 13:105–113. 10.1111/j.1364-3703.2011.00729.x. 21726396 PMC6638701

[koag160-B25] Hyde LS et al 2018. Response of cell-wall composition and RNA-seq transcriptome to methyl-jasmonate in *Brachypodium distachyon* callus. Planta. 248:1213–1229. 10.1007/s00425-018-2968-9. 30094490 PMC6182315

[koag160-B26] Im JH et al 2024. Jasmonate activates secondary cell wall biosynthesis through MYC2-MYB46 module. Plant J. 117:1099–1114. 10.1111/tpj.16541. 37983636

[koag160-B27] Jacobs AK et al 2003. An *Arabidopsis* callose synthase, GSL5, is required for wound and papillary callose formation. Plant Cell. 15:2503–2513. 10.1105/tpc.016097. 14555698 PMC280557

[koag160-B28] Jiang J, Fredua-Agyeman R, Strelkov SE, Hwang SF. 2020. Suppression of canola (*Brassica napus*) resistance by virulent isolates of *Plasmodiophora brassicae* (clubroot) during primary infection. Plant Dis. 104:430–437. 10.1094/PDIS-03-19-0659-RE. 31794288

[koag160-B29] Jiang X et al 2025. Jasmonic acid-mediated cell wall biosynthesis pathway involved in pepper (*Capsicum annuum*) defense response to *Ralstonia solanacearum*. BMC Plant Biol. 25:804. 10.1186/s12870-025-06784-4. 40604413 PMC12218943

[koag160-B30] Jirage D et al 1999. Arabidopsis thaliana PAD4 encodes a lipase-like gene that is important for salicylic acid signaling. Proc Natl Acad Sci USA. 96:13583–13588. 10.1073/pnas.96.23.13583. 10557364 PMC23991

[koag160-B31] Jørgensen JH . 1992. Discovery, characterization and exploitation of Mlo powdery mildew resistance in barley. Euphytica. 63:141–152. 10.1007/BF00023919.

[koag160-B32] Koo AJ, Howe GA. 2009. The wound hormone jasmonate. Phytochemistry. 70:1571–1580. 10.1016/j.phytochem.2009.07.018. 19695649 PMC2784233

[koag160-B33] Kuginuki Y, Yoshikawa H, Hirai M. 1999. Variation in virulence of *Plasmodiophora brassicae* in Japan tested with clubroot-resistant cultivars of Chinese cabbage (*Brassica rapa* L. ssp pekinensis). Eur J Plant Pathol. 105:327–332. 10.1023/A:1008705413127.

[koag160-B34] Kusch S, Panstruga R. 2017. mlo-based resistance: an apparently universal “weapon” to defeat powdery mildew disease. Mol Plant Microbe Interact. 30:179–189. 10.1094/MPMI-12-16-0255-CR. 28095124

[koag160-B35] Liu L et al 2020a. Refining the life cycle of *Plasmodiophora brassicae*. Phytopathology. 110:1704–1712. 10.1094/PHYTO-02-20-0029-R. 32407251

[koag160-B36] Liu L et al 2020b. Comparing the infection biology of *Plasmodiophora brassicae* in clubroot susceptible and resistant hosts and non-hosts. Front Microbiol. 11:507036. 10.3389/fmicb.2020.507036. 33178139 PMC7596292

[koag160-B37] Liu X et al 2023. Balanced callose and cellulose biosynthesis in *Arabidopsis* quorum-sensing signaling and pattern-triggered immunity. Plant Physiol. 194:137–152. 10.1093/plphys/kiad473. 37647538 PMC10756761

[koag160-B38] Livak KJ, Schmittgen TD. 2001. Analysis of relative gene expression data using real-time quantitative PCR and the 2−ΔΔCT method. Methods. 25:402–408. 10.1006/meth.2001.1262. 11846609

[koag160-B39] Love MI, Huber W, Anders S. 2014. Moderated estimation of fold change and dispersion for RNA-seq data with DESeq2. Genome Biol. 15:550. 10.1186/s13059-014-0550-8. 25516281 PMC4302049

[koag160-B40] Lovelock DA et al 2016. Analysis of salicylic acid-dependent pathways in *Arabidopsis thaliana* following infection with *Plasmodiophora brassicae* and the influence of salicylic acid on disease. Mol Plant Pathol. 17:1237–1251. 10.1111/mpp.12361. 26719902 PMC6638340

[koag160-B41] Ludwig-Müller J et al 2015. A novel methyltransferase from the intracellular pathogen *Plasmodiophora brassicae* methylates salicylic acid. Mol Plant Pathol. 16:349–364. 10.1111/mpp.12185. 25135243 PMC6638400

[koag160-B42] Lux A et al 2011. Cadmium induces hypodermal periderm formation in the roots of the monocotyledonous medicinal plant *Merwilla plumbea*. Ann Bot. 107:285–292. 10.1093/aob/mcq240. 21118841 PMC3025738

[koag160-B43] Matsumoto E, Ueno H, Aruga D, Sakamoto K, Hayashida N. 2012. Accumulation of three clubroot resistance genes through marker-assisted selection in Chinese cabbage (ssp). J Jpn Soc Hortic Sci. 81:184–190. 10.2503/jjshs1.81.184.

[koag160-B44] McKenna S et al 2001. Noncovalent interaction between ubiquitin and the human DNA repair protein Mms2 is required for Ubc13-mediated polyubiquitination. J Biol Chem. 276:40120–40126. 10.1074/jbc.M102858200. 11504715

[koag160-B45] Nishimura MT et al 2003. Loss of a callose synthase results in salicylic acid-dependent disease resistance. Science. 301:969–972. 10.1126/science.1086716. 12920300

[koag160-B46] Patro R, Duggal G, Love MI, Irizarry RA, Kingsford C. 2017. Salmon provides fast and bias-aware quantification of transcript expression. Nat Methods. 14:417–419. 10.1038/nmeth.4197. 28263959 PMC5600148

[koag160-B47] Peng G et al 2015. A >2-year crop rotation reduces resting spores of *Plasmodiophora brassicae* in soil and the impact of clubroot on canola. Eur J Agron. 70:78–84. 10.1016/j.eja.2015.07.007.

[koag160-B48] Perez-de-Lis G et al 2024. Multimodal imaging analysis in silver fir reveals coordination in cellulose and lignin deposition. Plant Physiol. 195:2428–2442. 10.1093/plphys/kiae203. 38590143 PMC11213250

[koag160-B49] Pérez-López E et al 2018. Identification of *Plasmodiophora brassicae* effectors – A challenging goal. Virulence. 9:1344–1353. 10.1080/21505594.2018.1504560. 30146948 PMC6177251

[koag160-B50] Qin L et al 2021. The ARP2/3 complex, acting cooperatively with Class I formins, modulates penetration resistance in *Arabidopsis* against powdery mildew invasion. Plant Cell. 33:3151–3175. 10.1093/plcell/koab170. 34181022 PMC8462814

[koag160-B51] Ranathunge K, Schreiber L, Bi YM, Rothstein SJ. 2016. Ammonium-induced architectural and anatomical changes with altered suberin and lignin levels significantly change water and solute permeabilities of rice (*Oryza sativa* L.) roots. Planta. 243:231–249. 10.1007/s00425-015-2406-1. 26384983

[koag160-B52] Schindelin J et al 2012. Fiji: an open-source platform for biological-image analysis. Nat Methods. 9:676–682. 10.1038/nmeth.2019. 22743772 PMC3855844

[koag160-B53] Schwelm A et al 2015. The *Plasmodiophora brassicae* genome reveals insights in its life cycle and ancestry of chitin synthases. Sci Rep. 5:11153. 10.1038/srep11153. 26084520 PMC4471660

[koag160-B54] Serra O, Mähönen AP, Hetherington AJ, Ragni L. 2022. The making of plant armor: the periderm. Annu Rev Plant Biol. 73:405–432. 10.1146/annurev-arplant-102720-031405. 34985930

[koag160-B55] Soda N, Verma L, Giri J. 2018. CRISPR-Cas9 based plant genome editing: significance, opportunities and recent advances. Plant Physiol Biochem. 131:2–11. 10.1016/j.plaphy.2017.10.024. 29103811

[koag160-B56] Soneson C, Love MI, Robinson MD. 2015. Differential analyses for RNA-seq: transcript-level estimates improve gene-level inferences. F1000Res. 4:1521. 10.12688/f1000research.7563.1. 26925227 PMC4712774

[koag160-B57] Strelkov SE et al 2018. Virulence and pathotype classification of *Plasmodiophora brassicae* populations collected from clubroot resistant canola (*Brassica napus*) in Canada. Can. J. Plant Pathol. 40:284–298. 10.1080/07060661.2018.1459851.

[koag160-B58] Teixeira RT . 2022. Cork development: what lies within. Plants (Basel). 11:2671. 10.3390/plants11202671. 36297695 PMC9611905

[koag160-B59] Thangavel T, Tegg RS, Wilson CR. 2016. Toughing it out—disease-resistant potato mutants have enhanced tuber skin defenses. Phytopathology. 106:474–483. 10.1094/PHYTO-08-15-0191-R. 26780437

[koag160-B60] Truernit E et al 2008. High-resolution whole-mount imaging of three-dimensional tissue organization and gene expression enables the study of Phloem development and structure in *Arabidopsis*. Plant Cell. 20:1494–1503. 10.1105/tpc.107.056069. 18523061 PMC2483377

[koag160-B61] Tso HH, Galindo-Gonzalez L, Strelkov SE. 2021. Current and future pathotyping platforms for *Plasmodiophora brassicae* in Canada. Plants (Basel). 10:1446. 10.3390/plants10071446. 34371649 PMC8309272

[koag160-B62] Tu J . 2018. Investigation of the *Plasmodiophora brassicae* life cycle and its interactions with host plants [Doctoral dissertation]. Department of Biology (College of Graduate and Postdoctoral Studies, University of Saskatchewan: University of Saskatchewan), pp. 164.

[koag160-B63] Tu J, Qin L, Karunakaran C, Wei Y, Peng G. 2024. Lignin accumulation in cell wall plays a role in clubroot resistance. Front Plant Sci. 15:1401265. 10.3389/fpls.2024.1401265. 39109069 PMC11300216

[koag160-B64] Ursache R, Andersen TG, Marhavý P, Geldner N. 2018. A protocol for combining fluorescent proteins with histological stains for diverse cell wall components. Plant J. 93:399–412. 10.1111/tpj.13784. 29171896

[koag160-B65] van Schie CC, Takken FL. 2014. Susceptibility genes 101: how to be a good host. Annu Rev Phytopathol. 52:551–581. 10.1146/annurev-phyto-102313-045854. 25001453

[koag160-B66] Vatén A et al 2011. Callose biosynthesis regulates symplastic trafficking during root development. Dev Cell. 21:1144–1155. 10.1016/j.devcel.2011.10.006. 22172675

[koag160-B67] Vogel J, Somerville S. 2000. Isolation and characterization of powdery mildew-resistant *Arabidopsis* mutants. Proc Natl Acad Sci U S A. 97:1897–1902. 10.1073/pnas.030531997. 10677553 PMC26533

[koag160-B68] von Malek B, van der Graaff E, Schneitz K, Keller B. 2002. The *Arabidopsis* male-sterile mutant dde2-2 is defective in the ALLENE OXIDE SYNTHASE gene encoding one of the key enzymes of the jasmonic acid biosynthesis pathway. Planta. 216:187–192. 10.1007/s00425-002-0906-2. 12430030

[koag160-B69] Walerowski P et al 2018. Clubroot disease stimulates early steps of phloem differentiation and recruits SWEET sucrose transporters within developing galls. Plant Cell. 30:3058–3073. 10.1105/tpc.18.00283. 30413655 PMC6354258

[koag160-B70] Wallenhammar AC . 1996. Prevalence of *Plasmodiophora brassicae* in a spring oilseed rape growing area in central Sweden and factors influencing soil infestation levels. Plant Pathol. 45:710–719. 10.1046/j.1365-3059.1996.d01-173.x.

[koag160-B71] Wang L et al 2019. *Arabidopsis* UBC13 differentially regulates two programmed cell death pathways in responses to pathogen and low-temperature stress. New Phytol. 221:919–934. 10.1111/nph.15435. 30218535

[koag160-B72] Wang W et al 2023. WeiTsing, a pericycle-expressed ion channel, safeguards the stele to confer clubroot resistance. Cell. 186:2656–2671.e18. 10.1016/j.cell.2023.05.023. 37295403

[koag160-B73] Wang ZP et al 2015. Egg cell-specific promoter-controlled CRISPR/Cas9 efficiently generates homozygous mutants for multiple target genes in *Arabidopsis* in a single generation. Genome Biol. 16:144. 10.1186/s13059-015-0715-0. 26193878 PMC4507317

[koag160-B74] Wen R et al 2014. UBC13, an E2 enzyme for Lys63-linked ubiquitination, functions in root development by affecting auxin signaling and Aux/IAA protein stability. Plant J. 80:424–436. 10.1111/tpj.12644. 25142088

[koag160-B75] Wen R et al 2020. Quantification of *Plasmodiophora brassicae* resting spores in soils using droplet digital PCR (ddPCR). Plant Dis. 104:1188–1194. 10.1094/PDIS-03-19-0584-RE. 32065569

[koag160-B76] Wu Y et al 2025. Inactivation of β-1,3-glucan synthase-like 5 confers broad-spectrum resistance to *Plasmodiophora brassicae* pathotypes in cruciferous plants. Nat Genet. 57:2302–2312. 10.1038/s41588-025-02306-y. 40890362 PMC12425816

[koag160-B77] Wunderling A et al 2018. A molecular framework to study periderm formation in *Arabidopsis*. New Phytol. 219:216–229. 10.1111/nph.15128. 29611875

[koag160-B78] Xie B, Wang X, Zhu M, Zhang Z, Hong Z. 2011. Cals7 encodes a callose synthase responsible for callose deposition in the phloem. Plant J. 65:1–14. 10.1111/j.1365-313X.2010.04399.x. 21175885

[koag160-B79] Xu H et al 2024. The JA-to-ABA signaling relay promotes lignin deposition for wound healing in *Arabidopsis*. Mol Plant. 17:1594–1605. 10.1016/j.molp.2024.09.003. 39262116

[koag160-B80] Xu L et al 2018. Jasmonic acid-mediated aliphatic glucosinolate metabolism is involved in clubroot disease development in *Brassica napus* L. Front Plant Sci. 9:750. 10.3389/fpls.2018.00750. 29922320 PMC5996939

[koag160-B81] Zaidi SS, Mukhtar MS, Mansoor S. 2018. Genome editing: targeting susceptibility genes for plant disease resistance. Trends Biotechnol. 36:898–906. 10.1016/j.tibtech.2018.04.005. 29752192

[koag160-B82] Zaveská Drábková L, Honys D. 2017. Evolutionary history of callose synthases in terrestrial plants with emphasis on proteins involved in male gametophyte development. PLoS One. 12:e0187331. 10.1371/journal.pone.0187331. 29131847 PMC5683620

[koag160-B83] Zhang P, Jackson E, Li X, Zhang Y. 2025. Salicylic acid and jasmonic acid in plant immunity. Hortic Res. 12:uhaf082. 10.1093/hr/uhaf082. 40343347 PMC12058309

[koag160-B84] Zhou N, Tootle TL, Tsui F, Klessig DF, Glazebrook J. 1998. PAD4 functions upstream from salicylic acid to control defense responses in *Arabidopsis*. Plant Cell. 10:1021–1030. 10.1105/tpc.10.6.1021. 9634589 PMC144042

[koag160-B85] Zhou Y et al 2002. Control of petal and pollen development by the plant cyclin-dependent kinase inhibitor ICK1 in transgenic Brassica plants. Planta. 215:248–257. 10.1007/s00425-002-0752-2. 12029474

