# Supplementary material for: Loss of PMR4 callose synthase triggers jasmonic acid–dependent resistance to the clubroot disease in Arabidopsis and Brassica napus
Source: Plant Cell. 2026 Jun 3;38(6):koag160. doi: 10.1093/plcell/koag160 (PMC13291821; doi:10.1093/plcell/koag160)
Supplement: koag160_Supplementary_Data [file koag160_supplementary_data.zip › PMR4-clubroot Supplementary tables.docx]

**SUPPLEMENTARY TABLES**

**Table S1.** *Arabidposis* mutant lines used in this study

| **Mutant line** | **Germplasm** | **Type of mutation** | **Sources** |
| --- | --- | --- | --- |
| **Mutant lines screened** | | | |
| *snc1-r1* | N/A | Substitution | (Zhang et al., 2003) |
| *snc1-1* | CS69908 | EMS-induced point mutation | ABRC |
| *dnd1-1* | CS6523 | Substitution | ABRC |
| *cpr1-1* | SALK_045148 | T-DNA insertion | ABRC |
| *npr1-3* | CS3802 | Substitution | ABRC |
| *ics1-9* | sid2-1 | Substitution | ABRC |
| *edr2* | N657975 | T-DNA insertion | ABRC |
| *pmr4-1* | CS3858 | Substitution | ABRC |
| *rabA4c-1* | SALK_005267 | T-DNA insertion | ABRC |
| *mlo2-5/6-2/12-1* | CS66569 | T-DNA insertion | ABRC |
| *bri1-5* | CS6126 | T-DNA insertion | ABRC |
| *cpn1-1* | N687486 | T-DNA insertion | ABRC |
| *sweet11/12* | CS68845 | T-DNA insertion | ABRC |
| *sweet11/12/15* | CS68998 | T-DNA insertion | ABRC |
| *abp1-1* | CS69045 | Indel | ABRC |
| *tir1-1* | CS3798 | T-DNA insertion | ABRC |
| *tir1-1 afb2-3 afb4-8 afb5-5* | CS69648 | T-DNA insertion | ABRC |
| *tir1-1 afb1-3 afb2-3 afb3-4* | CS69654 | T-DNA insertion | ABRC |
| *aux1-7* | CS3074 | Substitution | ABRC |
| *axr2-1* | CS3077 | Substitution | ABRC |
| *gh3.17* | SALK_050597.56.00.x | T-DNA insertion | ABRC |
| *gh3.5* | SALK_014376.54.75.X | T-DNA insertion | ABRC |
| *pin2* | CS8058 | Substitution | ABRC |
| *ubc13a-1/b-1* | WiscDsLox323H12, SALK_047381 | T-DNA insertion | This study |
| *rglg1/2* | rglg1 rglg2 | T-DNA insertion | (Yin et al., 2007) |
| *otu1* | SALK_058652.53.20.X | T-DNA insertion | ABRC |
| **Additional mutant lines used in this study** | | | |
| *NahG* | N/A | transgenic line | Lab stock |
| *pmr4-1 NahG* | CS67159 | transgenic line | ABRC |
| *pad4-1* | CS3806 | Substitution | ABRC |
| *pmr4-1 pad4-1* | CS67158 | Substitution | ABRC |
| *dde2-2* | CS56993 | Transposon insertion | ABRC |
| *pmr4-1 dde2-2* | CS72169 | Transposon insertion | ABRC |
| *gsl5-1* | GABI-KAT 089H05 | T-DNA insertion | NASC |

**Table S2.** Oligonucleotides used in this study

| **Primer name** | **Primer sequence (5' - 3')** | **Primer use** |
| --- | --- | --- |
| *pmr4-1*-NheI-F | TTACCAGCCCAACCAATTTC | Genotyping *pmr4-1* |
| *pmr4-1*-NheI-R | AGATCAGGGACATGGGACAG | Genotyping *pmr4-1* |
| *NahG*-F | GCCTTAGCACTGGAACTCTG | Genotyping *NahG* |
| *NahG*-R | TCGGTGAACAGCACTTGCAC | Genotyping *NahG* |
| *pad4*-F | GCGATGCATCAGAAGAG | Genotyping *pad4-1* |
| *pad4*-R | TTAGCCCAAAAGCAAGTATC | Genotyping *pad4-1* |
| *gsl5-1*-F | GGAAATCCCATCCTCAGTCTC | Genotyping *gsl5-1* |
| *gsl5-1*-R | TGGTAACAAGTTCAAGGACGG | Genotyping *gsl5-1* |
| *dde2-2Bsf*UI*-*F | GACACGAACCGGATCCAAAG | Genotyping *dde2-2* |
| *dde2-2Bsf*UI*-*R | GCCGAAATCCGCTTTCCCTTTA | Genotyping *dde2-2* |
| *dnd1-1* F | TCTAGAGAAGTCCGTCCATCGAA | Genotyping *dnd1-1* |
| *dnd1-1* R | TCTAGAGCGATCTTTGAGGTTTGCTC | Genotyping *dnd1-1* |
| *edr2*-F | GCTTTTGGGTTCGTTAATGTG | Genotyping *edr2* |
| *edr2*-R | TAACCATTTTGGCACTGAAGG | Genotyping *edr2* |
| *cpn1*-F | CACCAAAAAGGGGTCCTAAAG | Genotyping *cpn1* |
| *cpn1*-R | TCAAGTCCAAAGGTTTCAACG | Genotyping *cpn1* |
| *bri1*-F | TATTATAGCGGCAGTTGGTGG | Genotyping *bri1-5* |
| *bri1*-R | TTAAGATATCATCATCGGCGG | Genotyping *bri1-5* |
| *sweet11*-F | CCGAAGAGTAATGTGACCACG | Genotyping *sweet11* |
| *sweet11*-R | TGAAGTGGGTGCTTTTGTTTC | Genotyping *sweet11* |
| *sweet12*-F | ATGCAGGCCAACGTTCTATAG | Genotyping *sweet12* |
| *sweet12*-R | TCAAAGGCCAAAGCAATATACC | Genotyping *sweet12* T-DNA |
| *sweet15*-F | CGTTATCTAACTGACGGCGAC | Genotyping *sweet15* |
| *sweet15*-R | CAAGTCTCTGTACTCGGCTGG | Genotyping *sweet15* |
| *cpr1*-F | TTTCGTAAATTTTTACACAAAATCG | Genotyping *cpr1-1* |
| *cpr1*-R | TGTGAGTAGCCTTGTCTTGGG | Genotyping *cpr1-1* |
| *pin2*-F | CTAACACGTTGGTAATGGGAATC | Genotyping *pin2* |
| *pin2*-R | TGTCGTGAGGAGGAATAGAAACTT | Genotyping *pin2* |
| *aux1-7*-F | TCTTTCTTGTAGAATGCGGCG | Genotyping *aux1-7* |
| *aux1-7*-R | TCAAAGACGGTGGTGTAAAGC | Genotyping *aux1-7* |
| LBb1.3 | ATTTTGCCGATTTCGGAAC | SALK lines T-DNA primer |
| GABI-8474-LB | ATAATAACGCTGCGGACATCTACATTTT | GABI lines T-DNA primer |
| WiscDsLox-P745 | AACGTCCGCAATGTGTTATTAAGTTGTC | WiscDsLox lines T-DNA primer |
| Spm32 | TACGAATAAGAGCGTCCATTT TAGAGTGA | SM lines T-DNA primer |
| LB3 | TAGCATCTGAATTTCATAACCAATCTCGATACAC | SAIL lines T-DNA primer |
| *qAtUBQ10*-F | TCCAGGATAAGGAGGGCAT | qPCR of *AtUBQ10* |
| *qAtUBQ10*-R | CGTCTTTCCCGTTAGGGTT | qPCR of *AtUBQ10* |
| *qPR1*-F | ACGGGGAAAACTTAGCCTGG | RT-qPCR of *PR1* |
| *qPR1*-R | TTGGCACATCCGAGTCTCAC | RT-qPCR of *PR1* |
| *qPR2*-F | GCTTCCTTCTTCAACCACACAGC | RT-qPCR of *PR2* |
| *qPR2*-R | CGTTGATGTACCGGAATCTGAC | RT-qPCR of *PR2* |
| *qPR5*-F | TGCAAGAGTGCCTGTGAGAG | RT-qPCR of *PR5* |
| *qPR5*-R | TCCGGTACAAGTGAAGGTGC | RT-qPCR of *PR5* |
| *qPAD4*-F | GTGGTTGGATGAGGCGAGAA | RT-qPCR of *PAD4* |
| *qPAD4*-R | TTCAGATTCGCCTCCCACAC | RT-qPCR of *PAD4* |
| *qEDS1*-F | ACCTAACCGAGCGCTATCAC | RT-qPCR of *EDS1* |
| *qEDS1*-R | TTGTCCGGATCGAAGAAATC | RT-qPCR of *EDS1* |
| *qSAG101*-F | CCATGTTGTCTCTGGCGGAT | RT-qPCR of *SAG101* |
| *qSAG101*-R | ACCGATCTTGACCTCCTTGC | RT-qPCR of *SAG101* |
| *qDDE2*-F | CACGATGGGAGCGATTGAGA | RT-qPCR of *DDE2* |
| *qDDE2*-R | TCGTCGCCAACGGTTGATAA | RT-qPCR of *DDE2* |
| *qVSP1*-F | CACTGTCGAGAATCTCAAGG | RT-qPCR of *VSP1* |
| *qVSP1*-R | ACCAAATCAGCCCATTGGTC | RT-qPCR of *VSP1* |
| *qVSP2*-F | TCATACTCAGTGACCGTTGG | RT-qPCR of *VSP2* |
| *qVSP2*-R | TGTACACCACTTGCCTCAAG | RT-qPCR of *VSP2* |
| *qLOX2*-F | AACTACGATTGCATGGGTCA | RT-qPCR of *LOX2* |
| *qLOX2*-R | TCGGTTGGGAAAGTATCCTC | RT-qPCR of *LOX2* |
| *qTHI2.1*-F | CTGCCCTTCCAACCAAGCTA | RT-qPCR of *THI2.1* |
| *qTHI2.1*-R | TGGCACCACACACAGAAGTT | RT-qPCR of *THI2.1* |
| *qJAR1*-F | AAGGTGACTCTTGTGTGACC | RT-qPCR of *JAR1* |
| *qJAR1*-R | GTGACATACTCATCTGGTCC | RT-qPCR of *JAR1* |
| *qJAZ1*-F | GCATGCAAGCCTGATGTCAAT | RT-qPCR of *JAZ1* |
| *qJAZ1*-R | TGCCTAGGAAACAGATTCGTC | RT-qPCR of *JAZ1* |
| *qDIR5*-F | AACCTGTGGCAAGAGCTCAA | RT-qPCR of *DIR5* |
| *qDIR5*-R | CTGTCCCTCCGACAACAGAC | RT-qPCR of *DIR5* |
| *qDIR13*-F | CGACACTTGGCTCTCTTGGT | RT-qPCR of *DIR13* |
| *qDIR13*-R | GTAGCGATCCCACGAGTCAT | RT-qPCR of *DIR13* |
| *qPAL1*-F | ACACTGTCTCTCAAGTGGCG | RT-qPCR of *PAL1* |
| *qPAL1*-R | GAATCAACGGGTACGTTGCG | RT-qPCR of *PAL1* |
| *qGPAT5*-F | AGACTAGTGAAGCGGCCAAC | RT-qPCR of *GPAT5* |
| *qGPAT5*-R | TGACGATGATATGGCCACCG | RT-qPCR of *GPAT5* |
| *qFAR4*-F | TGATCTCGCGGCTAGCTACT | RT-qPCR of *FAR4* |
| *qFAR4*-R | TCAATCGCAACATCTGCAAAG | RT-qPCR of *FAR4* |
| *qLAC7*-F | ACACACCTTCAACGTACAAAACT | RT-qPCR of *LAC7* |
| *qLAC7*-R | TGGAAGATCCCATGCCAATGA | RT-qPCR of *LAC7* |
| *qLAC15*-F | ATGGGTCGAGATTAGCAGCG | RT-qPCR of *LAC15* |
| *qLAC15*-R | GCGAGTCTCGGAGTTTCCAA | RT-qPCR of *LAC15* |
| *DC1*-F | CCTAGCGCTGCATCCCATAT | qPCR of *Pb* biomass |
| *DC1m*-R | CGGCTAGGATGGTTCGAAA | qPCR of *Pb* biomass |
| *qPbActin1*-F | GGGACATCACCGACTACCTG | qPCR of *PbActin1* |
| *qPbActin1*-R | ACTGCTCCGAGTTGGACATC | qPCR of *PbActin1* |
| *qIFT172-1*-F | CAGCGCGATCGTTTCTCATTGA | RT-qPCR of *IFT172* |
| *qIFT172-1*-R | GACTTTGACGGGAACTTGTTGCA | RT-qPCR of *IFT172* |
| *BnActin2*-Probe | 5’-/5HEX/TGCTGGATT/ZEN/CTGGTGATGGTGTGT/ 3IABkFQ/-3’ | ddPCR of *BnActin2* |
| *Pb*-Probe | 5’-/56 FAM/CCATGTGAA/ZEN/CCGGTGACGTGCG/ 3IABkFQ/-3’ | ddPCR of *Pb* |
| *BnaPMR4*s-sgRNA3-F | ATTGACAACAGGAAACACGGCCAG | Primers used to assemble CRISPR/Cas9 vector |
| *BnaPMR4*s-sgRNA3-R | AAACCTGGCCGTGTTTCCTGTTGT |  |
| *BnaPMR4*s-sgRNA4-F | ATTGCTTTTGCTTGCACTGAGCC |  |
| *BnaPMR4*s-sgRNA4-R | AAACGGCTCAGTGCAAGCAAAAG |  |
| *BnaPMR4. C09*- sgRNA3&4-F | TATGGACTTGGGCGGCCA | Primers used to genotype transplants |
| *BnaPMR4. C09*- sgRNA3&4-R | GAACTGCTCACCCTGAATAGACACC |  |
| *BnaPMR4. A09*- sgRNA3&4-F | GGATTGGGGCGGCCG |  |
| *BnaPMR4. A09*- sgRNA3&4-R | TGAACTGCTCACCCTGAATAGAGACA |  |
| *BnaPMR4*s-sgRNA3&4-Seq-F | AGGTTGAGGCTAACAAGTTTGCG |  |

FAM: Fluorescein Amidite; HEX: Hexachloro-fluorescein; ZEN: Internal quencher; 3IABkFQ (3'-Iowa Black Fluorescence Quencher): 3'-end quencher

**References**

**Yin, X.J., Volk, S., Ljung, K., Mehlmer, N., Dolezal, K., Ditengou, F., Hanano, S., Davis, S.J., Schmelzer, E., Sandberg, G., Teige, M., Palme, K., Pickart, C., and Bachmair, A.** (2007). Ubiquitin lysine 63 chain forming ligases regulate apical dominance in Arabidopsis. Plant Cell **19,** 1898-1911.

**Zhang, Y., Goritschnig, S., Dong, X., and Li, X.** (2003). A gain-of-function mutation in a plant disease resistance gene leads to constitutive activation of downstream signal transduction pathways in suppressor of npr1-1, constitutive 1. Plant Cell **15,** 2636-2646.
