# Supplementary material for: Loss of PMR4 callose synthase triggers jasmonic acid–dependent resistance to the clubroot disease in Arabidopsis and Brassica napus
Source: Plant Cell. 2026 Jun 3;38(6):koag160. doi: 10.1093/plcell/koag160 (PMC13291821; doi:10.1093/plcell/koag160)
Supplement: koag160_Supplementary_Data [file koag160_supplementary_data.zip › PMR4-clubroot TPC Supplemental figures.docx]

**SUPPLEMENTARY FIGURES**

**
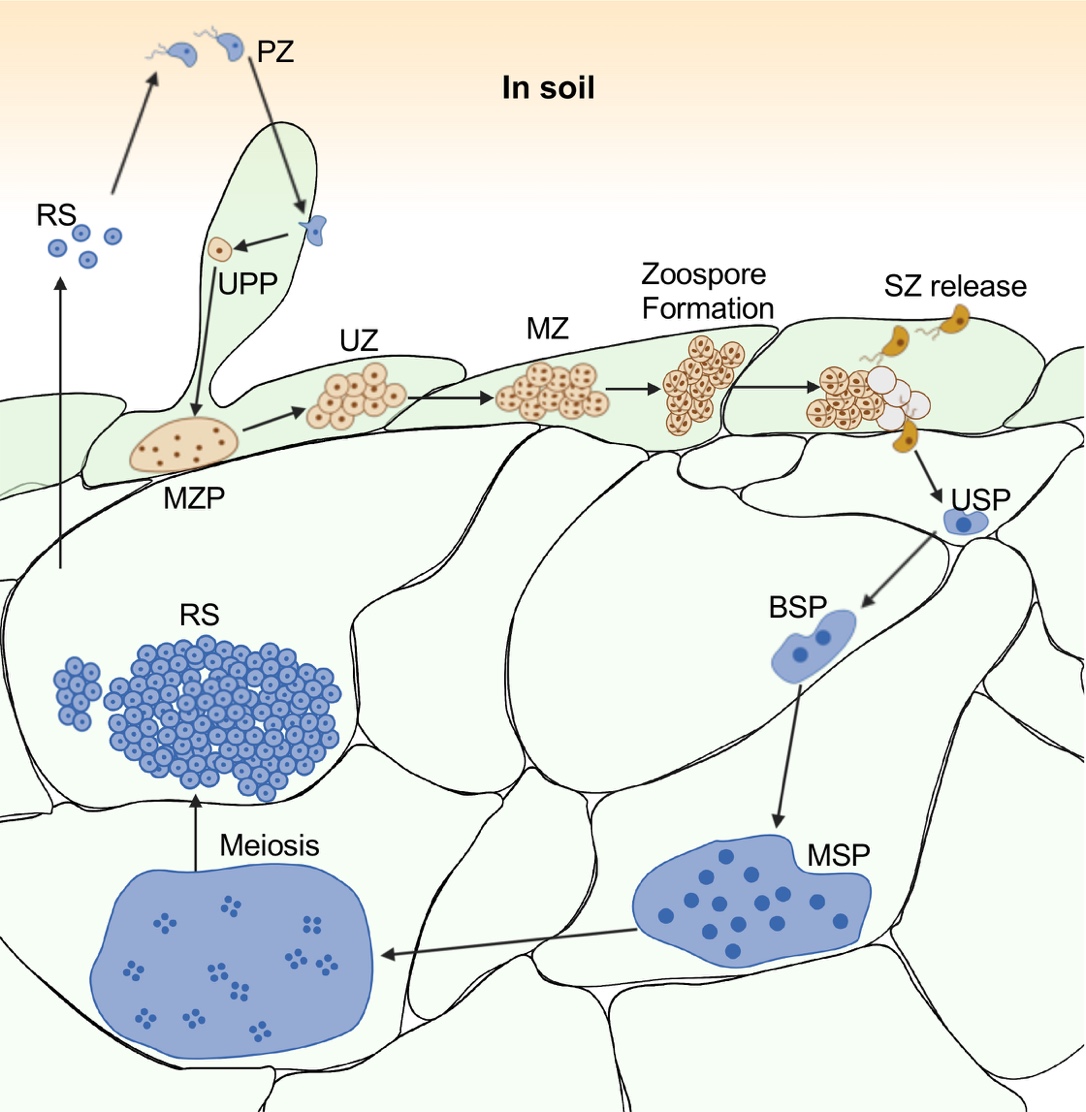
**

**Figure S1**. **The disease cycle of *Plasmodiophora brassicae****.*

The *in planta* life stages of *P. brassicae* involve the primary infection in host root hairs and epidermal cells, and the secondary infection in the cortical and stele tissues. The primary infection begins with resting spores (RS) in soil that germinate to produce primary zoospores (PZ) and further develop into uninucleate primary plasmodia (UPP), multinucleate zoosporangial plasmodia (MZP), uninucleate zoosporangia (UZ) and uninucleate secondary zoospores (MZ) (0-7 days post inoculation, dpi). Upon the release of secondary zoospores (SZ) into the soil or their penetration into the cortex and inner stele tissue, the secondary infection starts with uninucleate secondary plasmodia (USP) in cortical cells, which further develops into binucleate secondary plasmodia (BSP), multinucleate secondary plasmodia (MSP) and resting spores (6-24 dpi). The time point determination of each *P. brassicae* developmental stage is defined in the susceptible *A. thaliana* Col-0. Figure was generated by using BioRender (<https://app.biorender.com/>). This figure is to provide background information.

**
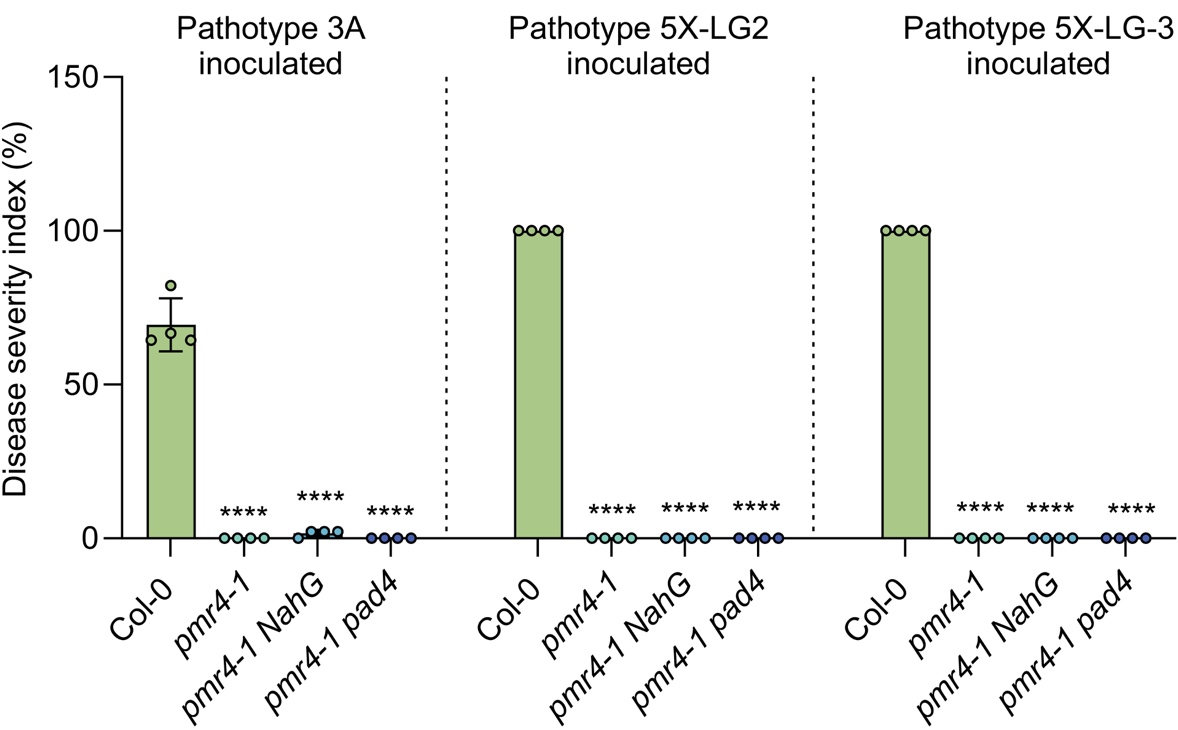
**

**Figure S2**. Genetic relationship between *Arabidopsis PMR4* and the SA pathway. *pmr4-1* resistance to different *Pb* pathotypes and its dependency on the functional SA pathway. Data are the average of four replicates, and each biological replicate contained 15 individual plants. Statistical differences were analyzed by one-way ANOVA, taking Col-0 as a control. Data represent means ± SD. ****, P≤0.0001. This figure expands and further supports Fig. 2a and b to additional *Pb* pathotypes.


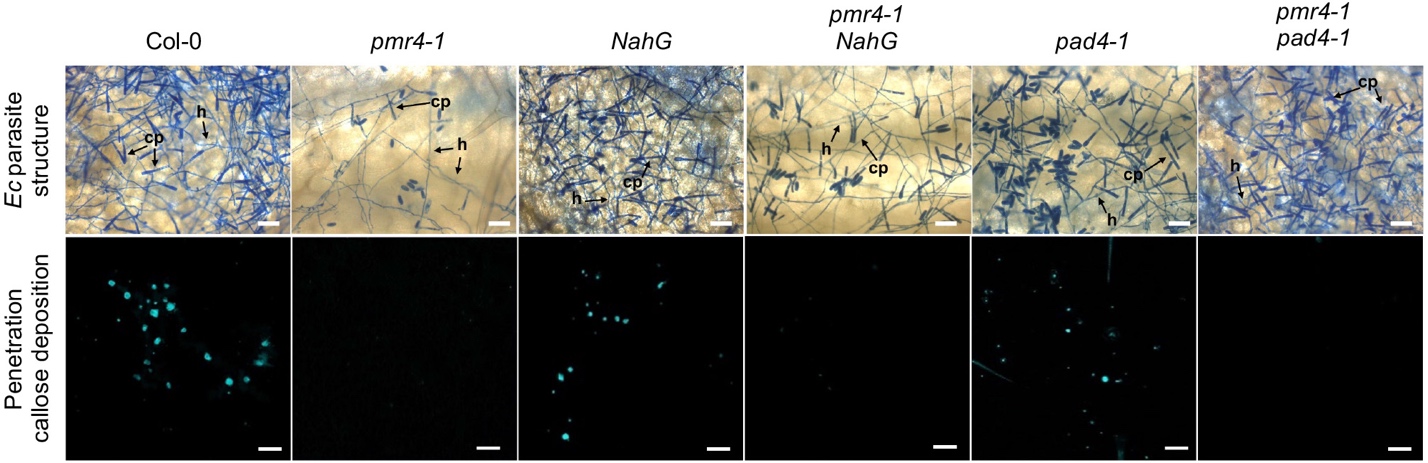


**Figure S3**. Microscopic analysis of the *Ec* pathogen in infected *Arabidopsis* leaves. Images in the upper row indicate hyphae growth and conidiophore production of *Ec* on leaf surfaces at 7 dpi stained with acidic aniline blue and observed with macroscopy. h, hyphae; cp, conidiophores. Scale bar =100 μm; Images from the lower row indicate *Ec*-induced penetration resistance-related papillae callose deposition stained with aniline blue at 2 dpi. Scale bar = 50 μm. This figure provides histological support to Fig. 2d and e.


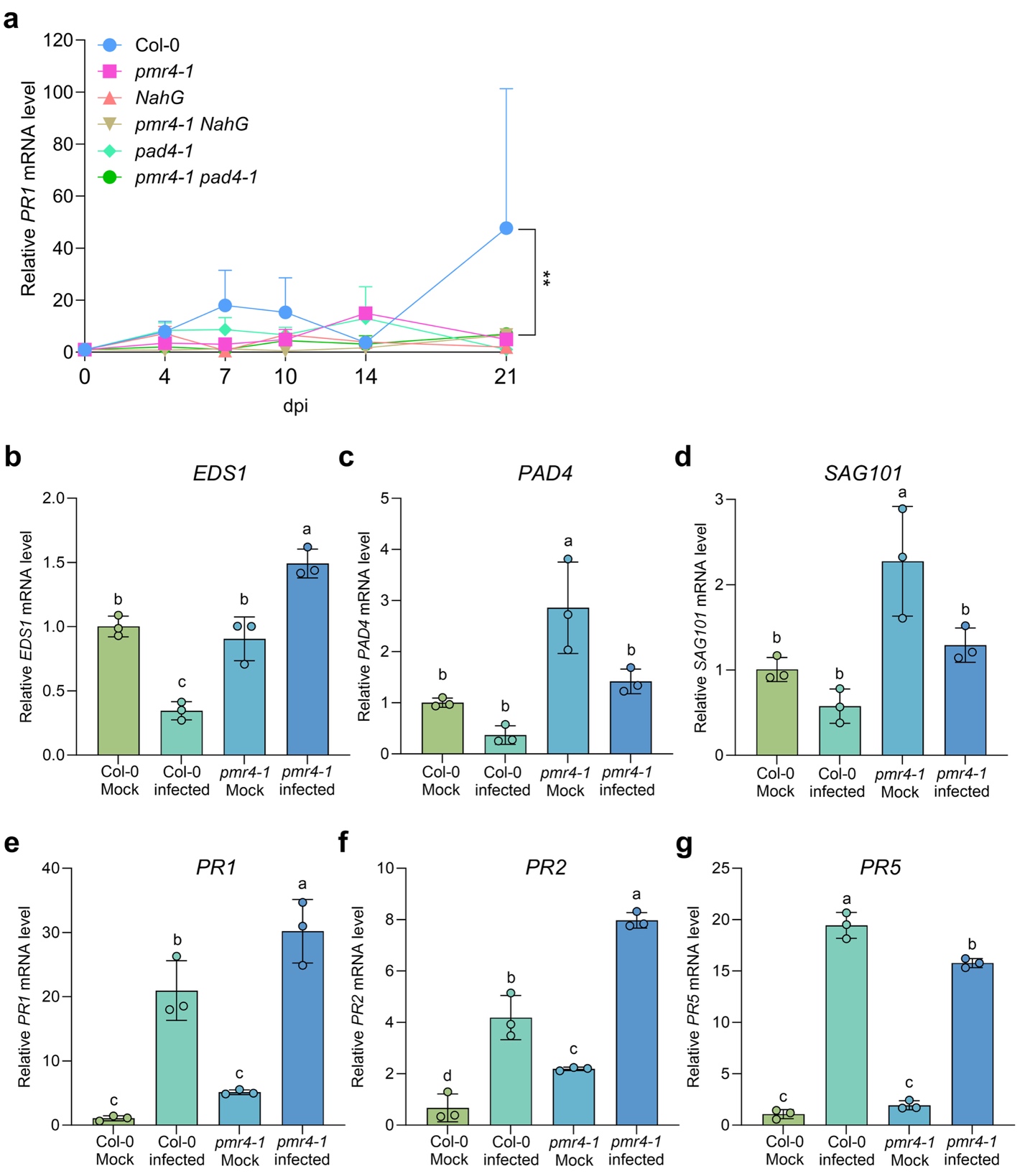


Figure S4

**Figure S4.** RT-qPCR analysis of genes involved in the SA-signaling. **a**) *PR1* transcript levels following inoculation with *Pb* pathotype 3H in roots from indicated genotypes for up to 21 dpi (n=3). All data were normalized to the expression of *AtUBQ10* and fold induction value of calculated relative to the expression level of inoculated plants at 0 dpi. Two-way ANOVA was performed. **, P≤0.01. Data represent means ± SD. **b**-**g**) qRT-PCR validation of RNA-seq data for genes involved in the SA-signaling. cDNA templates were generated from the same group of RNA-seq RNAs. *AtUBQ10* was used as an internal reference gene. The gene expression from mock Col-0 was selected as the control reference when calculating the relative expression profiles. One-way ANOVA was used to show the statistical significance. (α = 0.05). Letters represent the significant differences. Data represent means ± SD. n =3. Relative mRNA level of **b**) *EDS1*; **c**) *PAD4*; **d**) *SAG101*; **e**) *PR1*; **f**) *PR2*; and **g)** *PR5*. This figure aims to validate Fig. 2f.

**
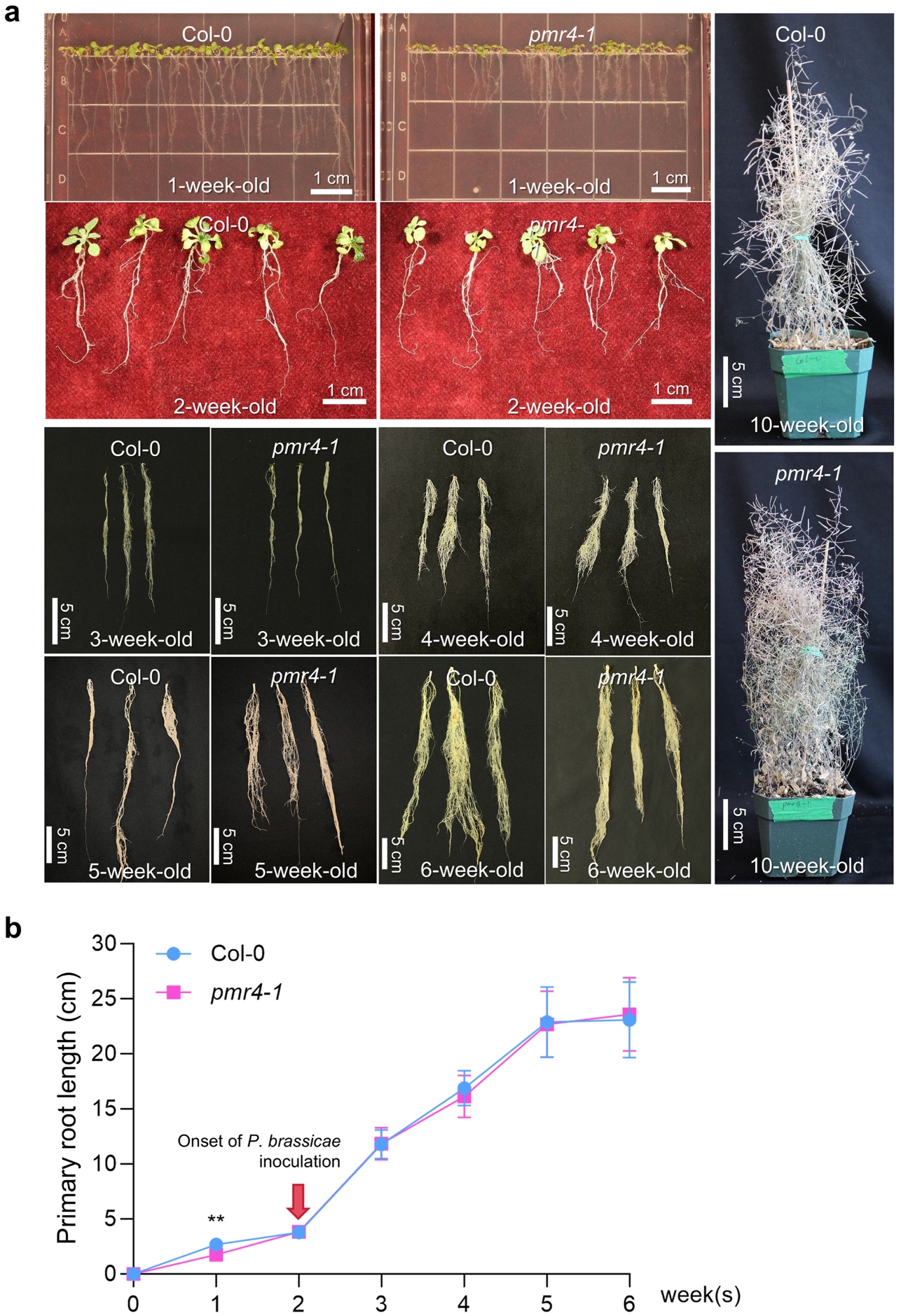
**

Figure S5

**Figure S5**. *pmr4-1* phenotypes under normal growth conditions in comparison to Col-0. **a**) Representative seedling and root phenotypes up to six-week growth and mature plants. Scale bars are as indicated. Primary root lengths for both lines were compared at the first six weeks of the whole life cycle. The mature plants were photographed at 10 weeks after seeding. **b**) Statistical analysis of primary root lengths of Col-0 and *pmr4-1* plants (n≥20). Data represent means ± SD. Statistical differences were analyzed by Two-way ANOVA, taking Col-0 as the control. **, P≤0.01. This figure is to support a claim that the *pmr4-1* mutation did not plant growth and development under uninfected conditions.


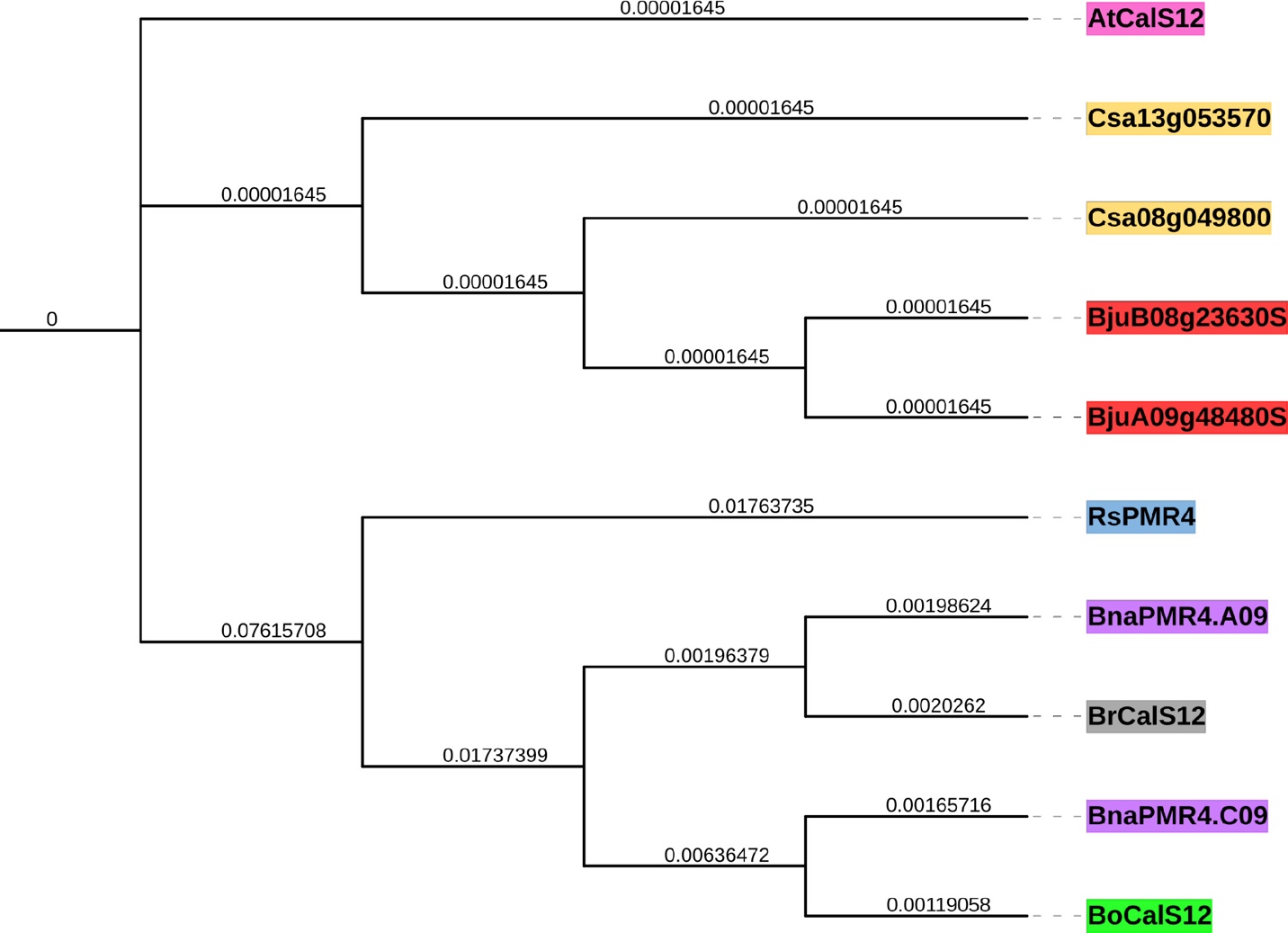


**Figure S6**. A phylogenetic tree of *PMR4/CalS12* orthologs in representative Brassicaceae crops. The *Arabidopsis* PMR4/CalS12 amino acid sequence was used as a query sequence for BLAST analysis against the EnsemblPlants protein database (https://plants.ensembl.org/Multi/Tools/Blast). Amino acid sequences were aligned and phylogenetic trees constructed by using MEGA 11.0 and iTOL (Letunic and Bork, 2021). Bootstrap levels are indicated on corresponding branches. *Raphanus sativus* (Rs, blue), *Arabidopsis thaliana* (At, pink), *B. napus* (Bna, purple), *B. rapa* (Br, grey), *B. camelina* (Csa, yellow), *B. juncea* (Bju, red) and *B. oleracea* (Bo, green). This figure is to support a notion that the *PMR4* gene is conserved in Brassicaceae crops.

Figure S3


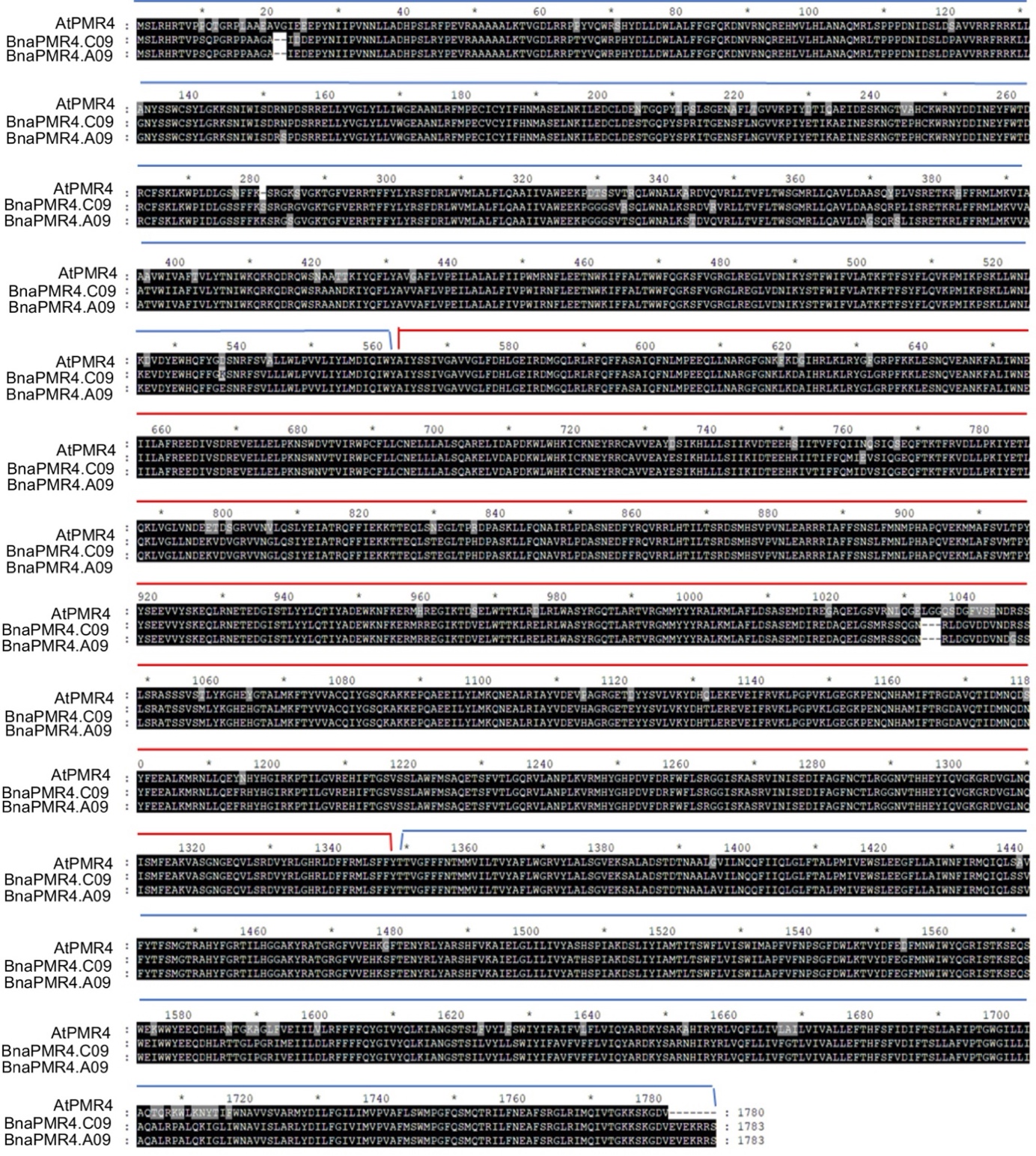


**Figure S7**. Amino acid sequence alignment of AtPMR4 and its two orthologs identified from the *B. napus* genome. The predicted amino acid sequences of AtPMR4, BnaPMR4.C09 and BnaPMR4.A09 were aligned by the MEGA 11.0 software. Blue lines indicate transmembrane domains at both N- (aa. 1-563) and C-termini (aa 1349-1780), while the red line shows putative catalytic regions (aa 564-1348), based on the AtPMR4 amino acid sequence. This figure is to justify the *BnPMR4* gene targeting strategy.

**
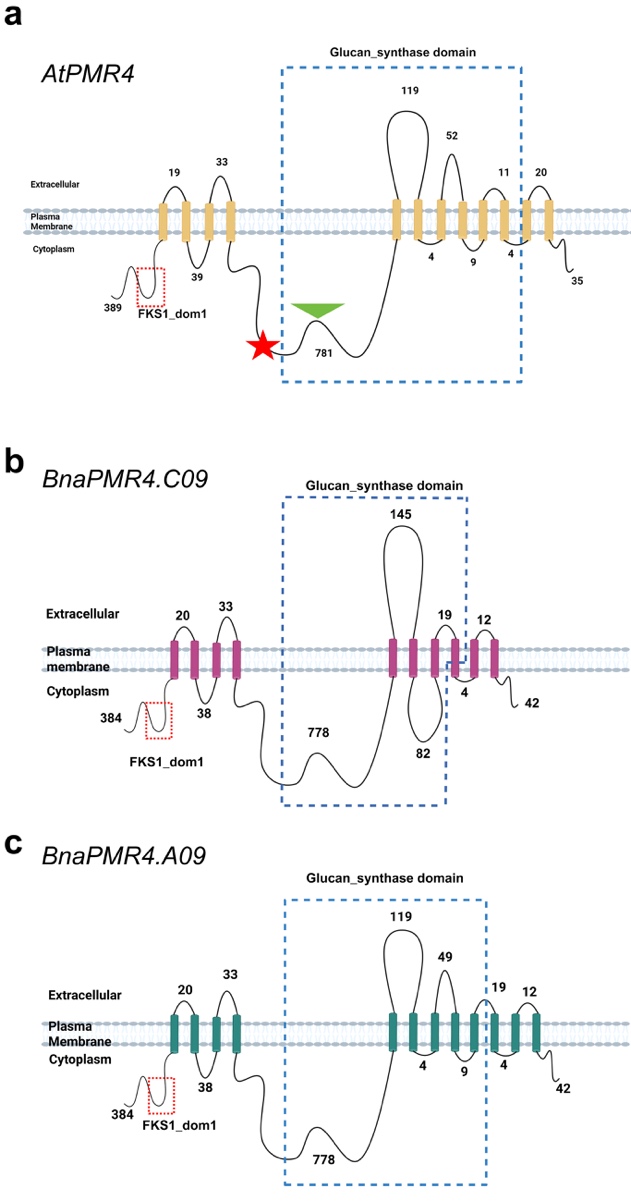
**

**Figure S8**. Predicted AtPMR4 (**a**), BnaPMR4.C09 (**b**) and BnaPMR4.A09 (**c**) protein topology. Topological structures were predicted by an online DeepTMHMM platform (Krogh et al., 2001). Conserved functional motifs or domains were predicted through an online Pfam database (Pfam: The protein families database in 2021: Mistry 2021). BnaPMR4.C09 and BnaPMR4.A09 are predicted to be polytopic integral membrane proteins with 10 and 12 transmembrane helices, respectively. Red and blue dash lined boxes mark the 1,3-beta-glucan synthase FKS1-like (FKS1_dom1) and 1,3-beta-glucan synthase (Glucan_synthase) domains, respectively. Amino acid sequences ‘Q9ZT82’, ‘A0A078IF26’ and ‘A0A078G9C0’ were used as inputs for both topological and functional domain predictions. The diagram was drawn in proportion by BioRender (https://app.biorender.com/). In AtPMR4 (A), a red asterisk indicates the nonsense mutation site in *pmr4-1*, and a green triangle indicates the T-DNA insertion site in *gsl5-1*. This figure is to justify the *BnPMR4* gene targeting strategy.

**
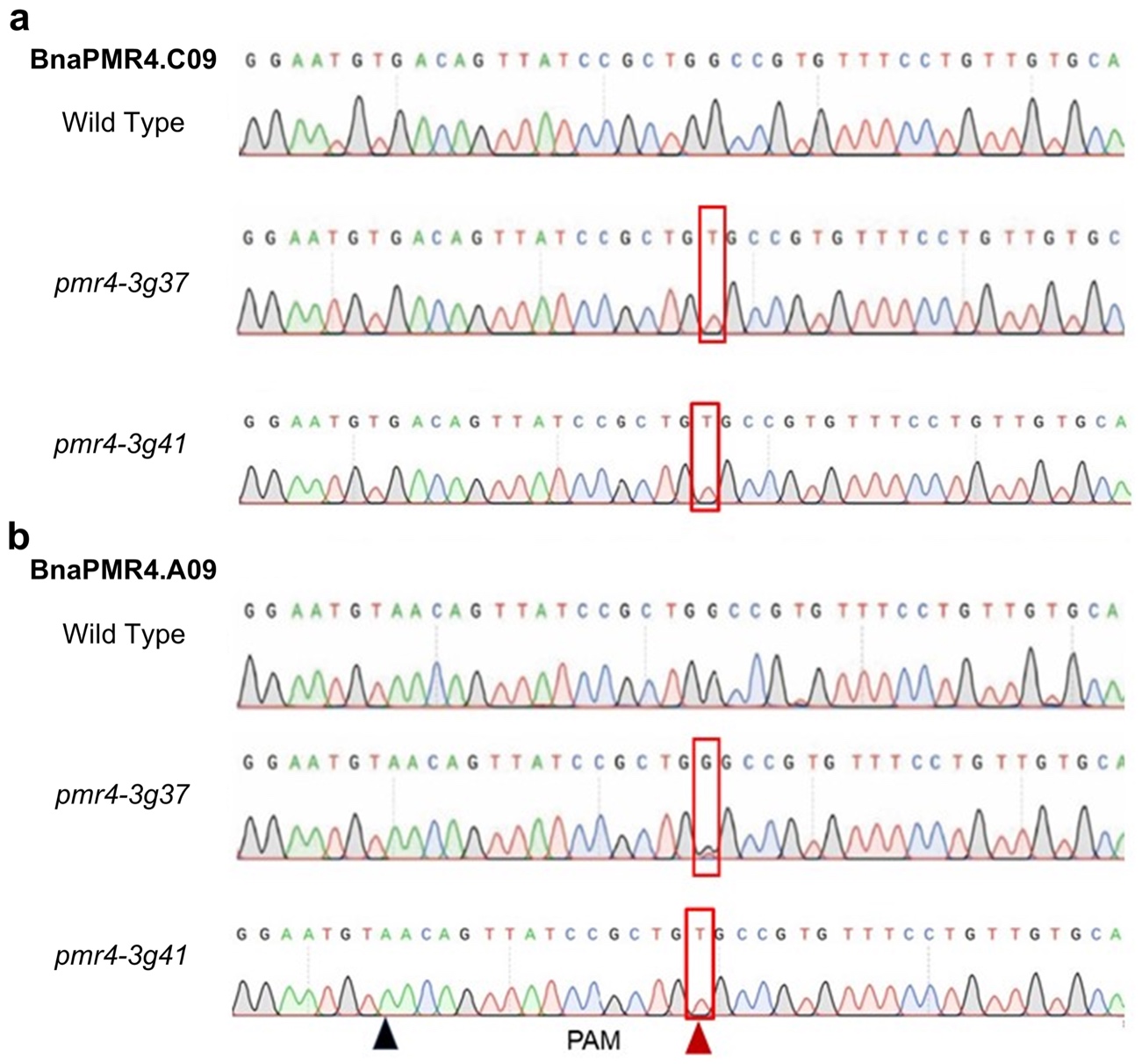
**

**Figure S9**. Histograms showing the nucleotide sequences of *pmr43g37* and *pmr4-3g41* T0 at *BnaPMR4* loci surrounding the sgRNA3 PAM sequence. **a**) At the *BnaPMR4.C09* locus. **b**) At the *BnaPMR4.A09* locus. The black arrowhead indicates single nucleotide variation between *BnaPMR4.C09* and *BnaPMR4.A09* within the sequenced region, which was used to distinguish the two genes. The red arrowhead points to CRISPR/Cas9-mediated single nucleotide insertions in red boxes in comparison to corresponding DH12075 wild-type sequences. PAM, protospacer adjacent motif. This figure is to demonstrate success in the *BnPMR4* gene editing.


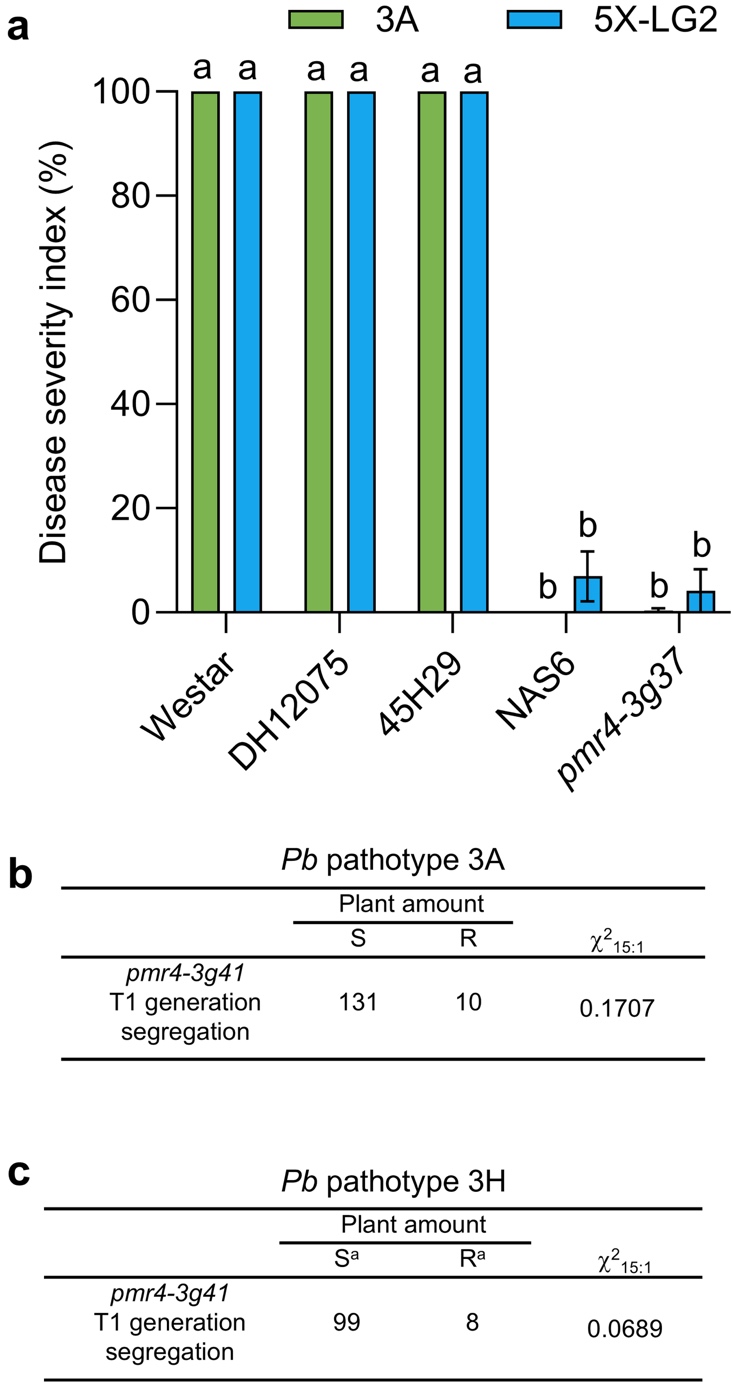


**Figure S10**. Assessment of *B. napus pmr4* mutants to the clubroot susceptibility infected by different *Pb* pathotypes. **a**) Quantitative measurement of clubroot disease severity against pathotypes 3A and 5X-LG2. Various canola strains including the gene-edited *pmr4-3g37* were inoculated with *Pb* pathotypes as indicated and DSI scored 28 dpi. Data represent for the means ± SD value obtained from 3 biological repeats, and each repeat contains at least 10 individual plants. Two-way ANOVA was performed, and different letters represent statistical significance (α = 0.05). **b**,**c**) Summary of heterozygous *pmr4-3g41* self-crossed T1 phenotypic segregants inoculated with *Pb* pathotypes 3A (B) and 3H (C) at 28 dpi and χ^2^ test for a two-gene independent segregation hypothesis. S, susceptible plants with disease; R, resistant plants exhibiting no disease symptoms. This figure is to extend phenotypic analyses as shown in Fig. 3.

**
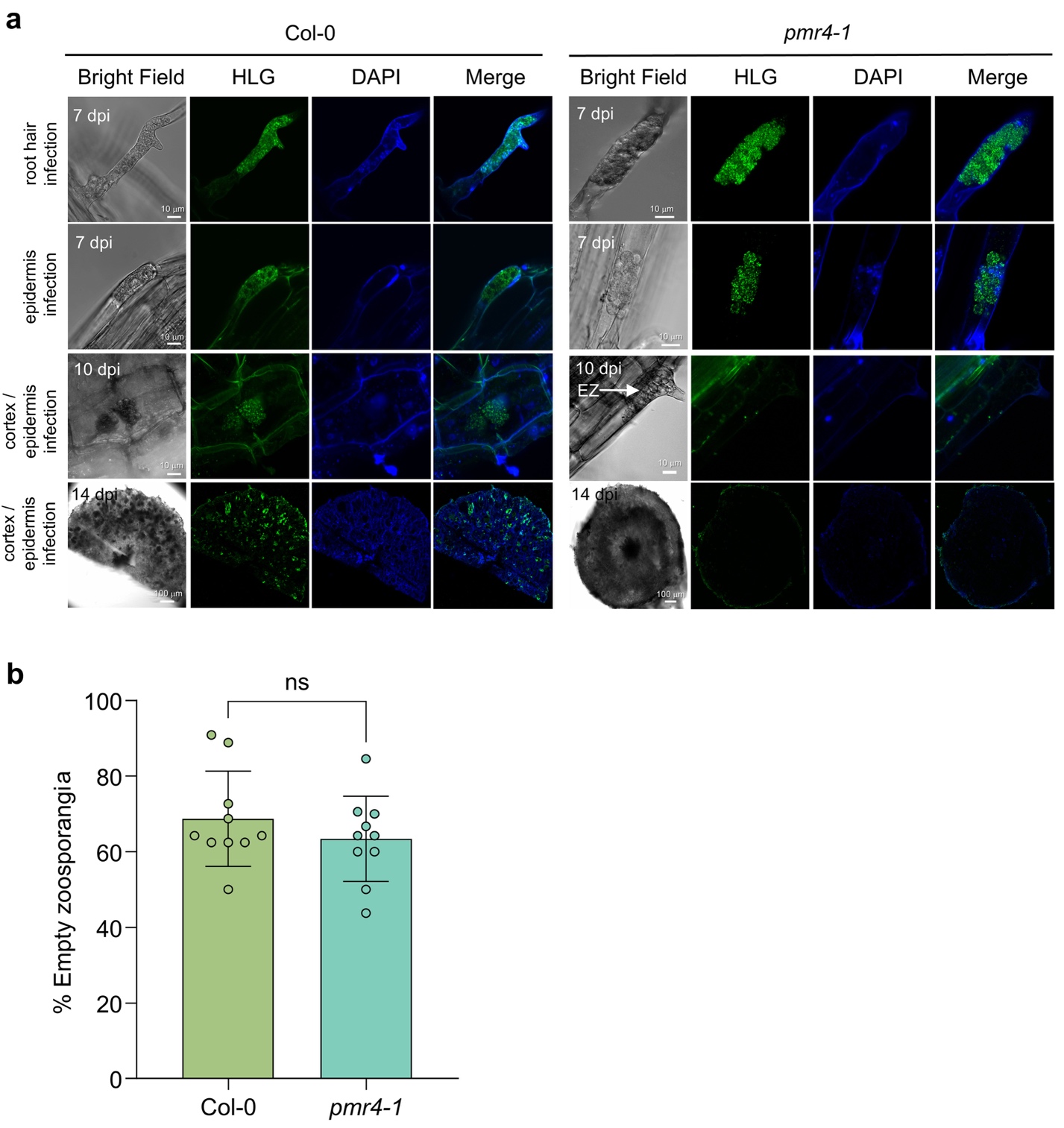
**

Figure S11

**Figure S11**. Microscopic analyses of *P. brassicae* disease cycles in *Arabidopsis* roots. **a**) Confocal laser scanning microscopy images of *Pb* development in *Arabidopsis* roots. Two-week-old seedlings of Col-0 and *pmr4-1* were inoculated with *Pb* pathotype 3H and roots were collected at 7, 10, and 14 dpi. Live cell dyes HCS LipidTox Green (HLG) and 4’ 6- diamidino-2-phenylinode (DAPI) were used to label *Pb* lipid droplets and nuclei, respectively. Empty zoosporangia (EZ) were indicated with white arrows. Scale bars = 10 μm. **b**) Proportion of empty zoosporangia detected from lateral roots proximate to the hypocotyl at 14 dpi. Statistical analysis was based on the images captured under 20x lens. EZ proportion was decided by determining the percentage of EZ out of the total *Pb* infection foci in each view. Data are means ± SD from 10 individual plants for each genotype. One-way ANOVA was used to calculate the statistical difference between genotypes. (α = 0.05). ns, no significance. This figure is to support a statement that the *pmr4-1* mutation did not affect *Pb* primary infection.

**
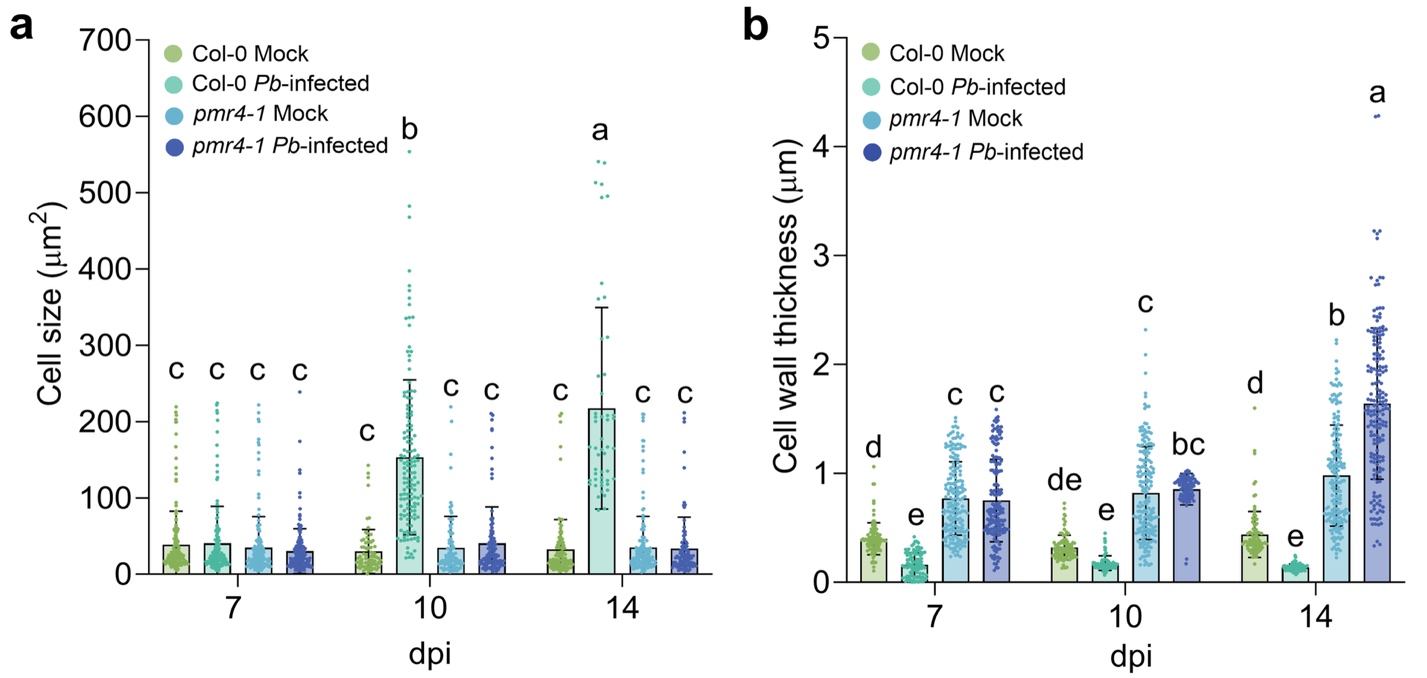
**

**Figure S12.** Quantitative analysis of mock and *P. brassicae*-infected Col-0 and *pmr4-1* roots. **a**) Average root cell sizes at 7, 10 and 14 dpi. Measurement was conducted using Fiji software. Statistics at each data point were based on 6 images, each of which usually contains 30 to 70 validated cells to be assessed. Dots on corresponding bars indicate individual values. Data represent means ± SD. Two-way ANOVA was performed to compare average cell size differences between Col-0 and *pmr4-1* at each time point, and different letters represent statistical significance (α = 0.05). **b**) Average root periderm cell wall thickness at 7, 10 and 14 dpi. Measurement was conducted using Fiji software. Statistics at each data point were based on 5 images, each of which was measured with 30 to 40 validated samples. Dots on corresponding bars indicate individual values. Data represent means ± SD. Two-way ANOVA was performed to compare average periderm cell wall thickness differences between Col-0 and *pmr4-1* at each time point, and different letters represent statistical significance (α = 0.05). This figure is to provide statistical data for Fig. 5d.


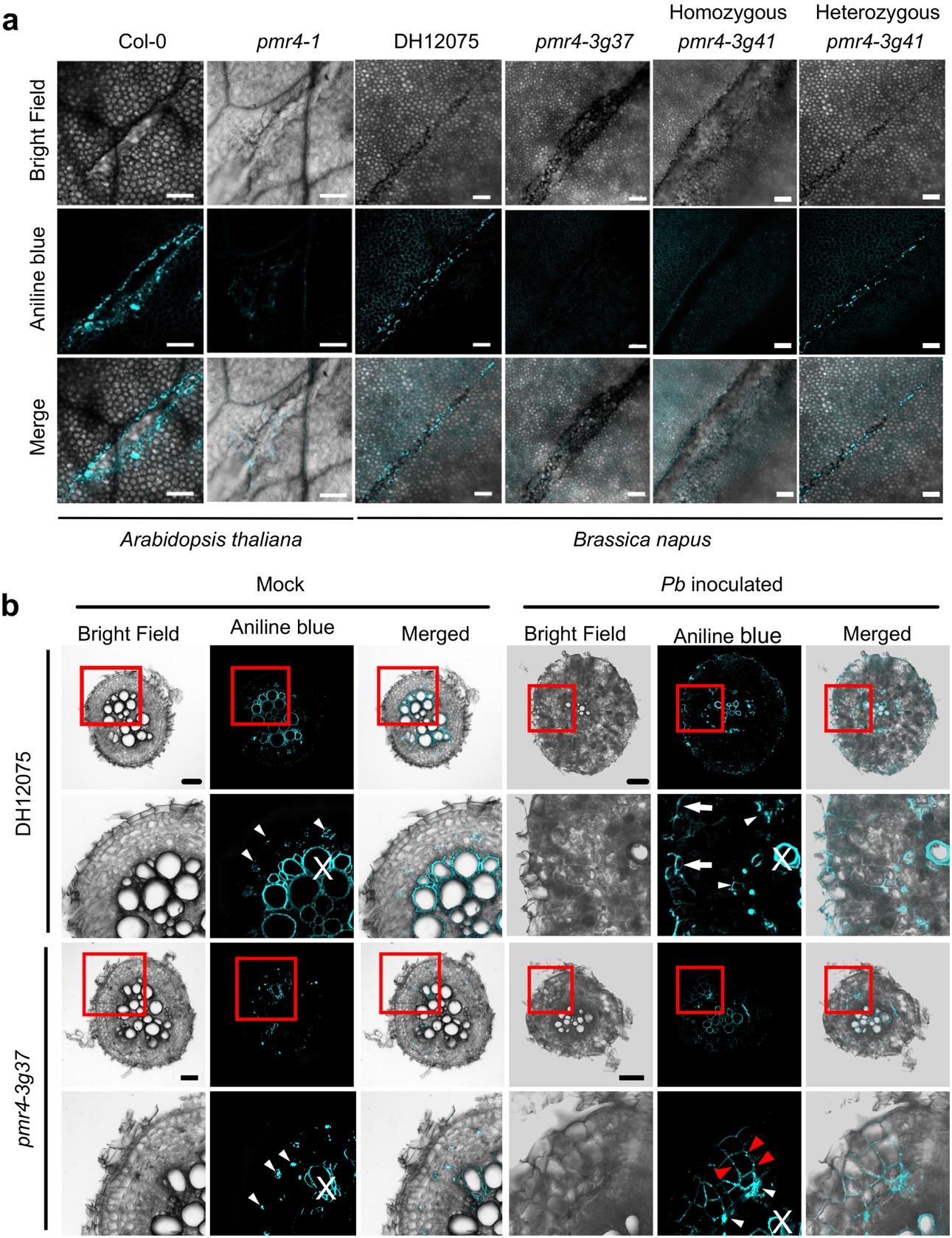


Figure S13

**Figure S13**. Wound- and *P. brassicae*-induced callose deposition in *B. napus*. **a**) Callose accumulation at wound sites in DH12075 and its *pmr4* mutant derivatives. *Arabidopsis* Col-0 and its *pmr4-1* mutant serve as references. Plant leaves were wounded by making cuts with a razor blade. The callose accumulation at the wound site was observed 24 hrs after the treatment by aniline blue fluorochrome staining. The heterozygous *pmr4-3g41* plant was from T0, and the homozygous *pmr4-3g41* plant was from genotype-confirmed T1 segregants. Scale bars = 10 µm. **b**) Callose deposition patterns in DH12075 and *pmr4-3g37* roots with or without *Pb* pathotype 3H infection. Lateral root samples were collected at 14 dpi, fixed, sliced into 50 μm transverse section and then stained with aniline blue. The boxed region in each upper row was enlarged to form the corresponding lower row. White arrowheads indicate sieve element or phloem constitutive callose deposition. Xylem lignin autofluorescence was labelled by white ‘X’. White arrows indicate *Pb*-induced callose deposition in DH12075. Red arrowheads indicate the punctate plasmodesmata (PD)-localized callose deposition induced by *Pb* infection in *pmr4-3g37*. Representative images were selected from samples collected from 5 individual plants, and 3 different sections were observed from each plant. Scale bars = 100 µm. This figure is to support observations made in Fig. 6 by using different methods and species.

**
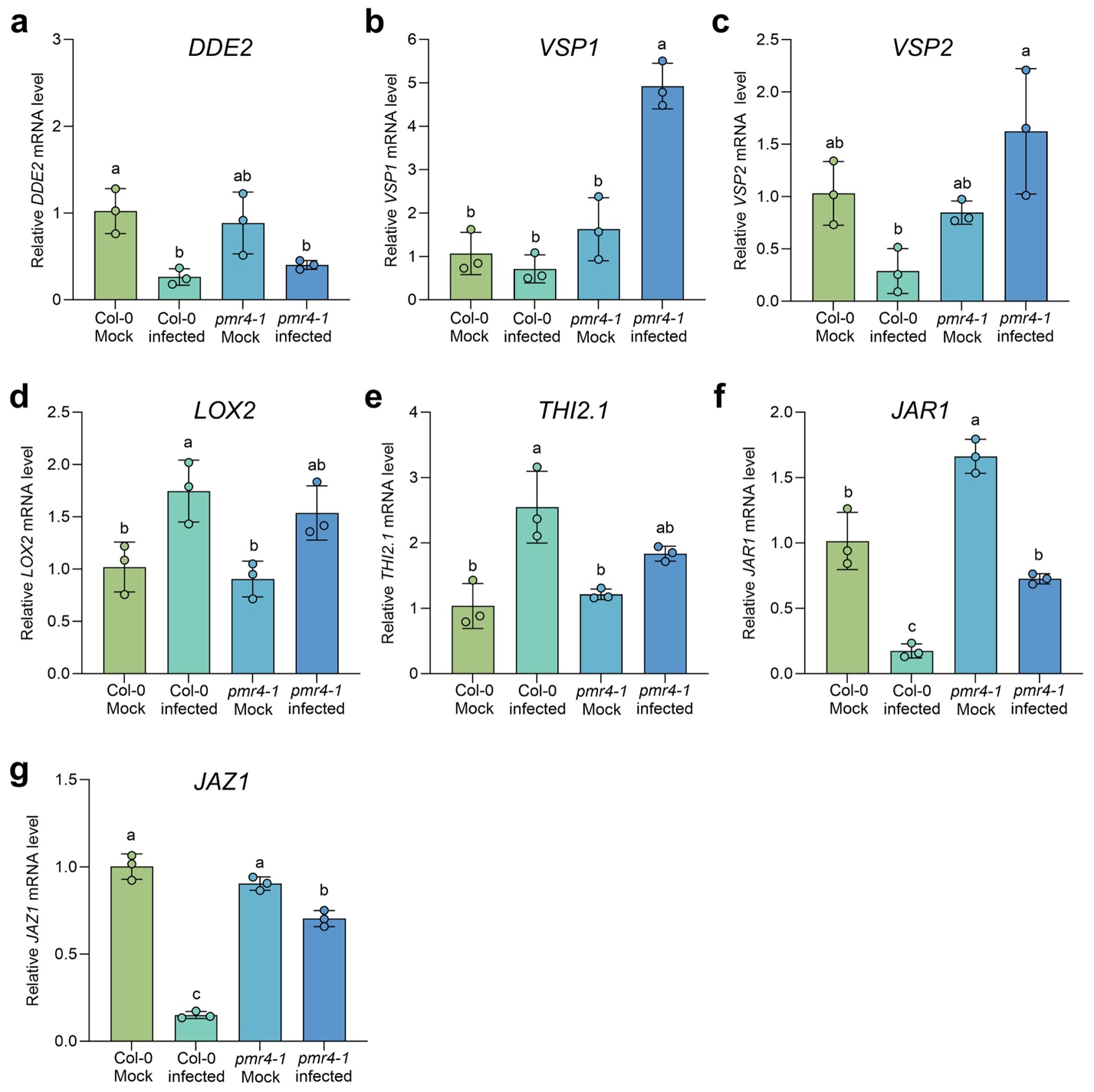
**

Figure S14

**Figure S14.** RT-qPCR validation of RNA-seq data for genes involved in the JA-signaling. Relative mRNA level of **a**) *DDE2*; **b**) *VSP1*; **c**) *VSP2*; **d**) *LOX2*; **e**) *THI2.1*; **f**) *JAR1*; and **g**) *JAZ1*. cDNA templates were generated from the same group of RNA-seq RNAs. *AtUBQ10* was used as an internal reference gene. The gene expression from mock Col-0 was selected as the control reference when calculating the relative expression profiles. One-way ANOVA was used to show the statistical significance. (α = 0.05). Letters represent the significant differences. Data = mean ± SD. n =3. This figure aims to validate Fig. 7e.

**
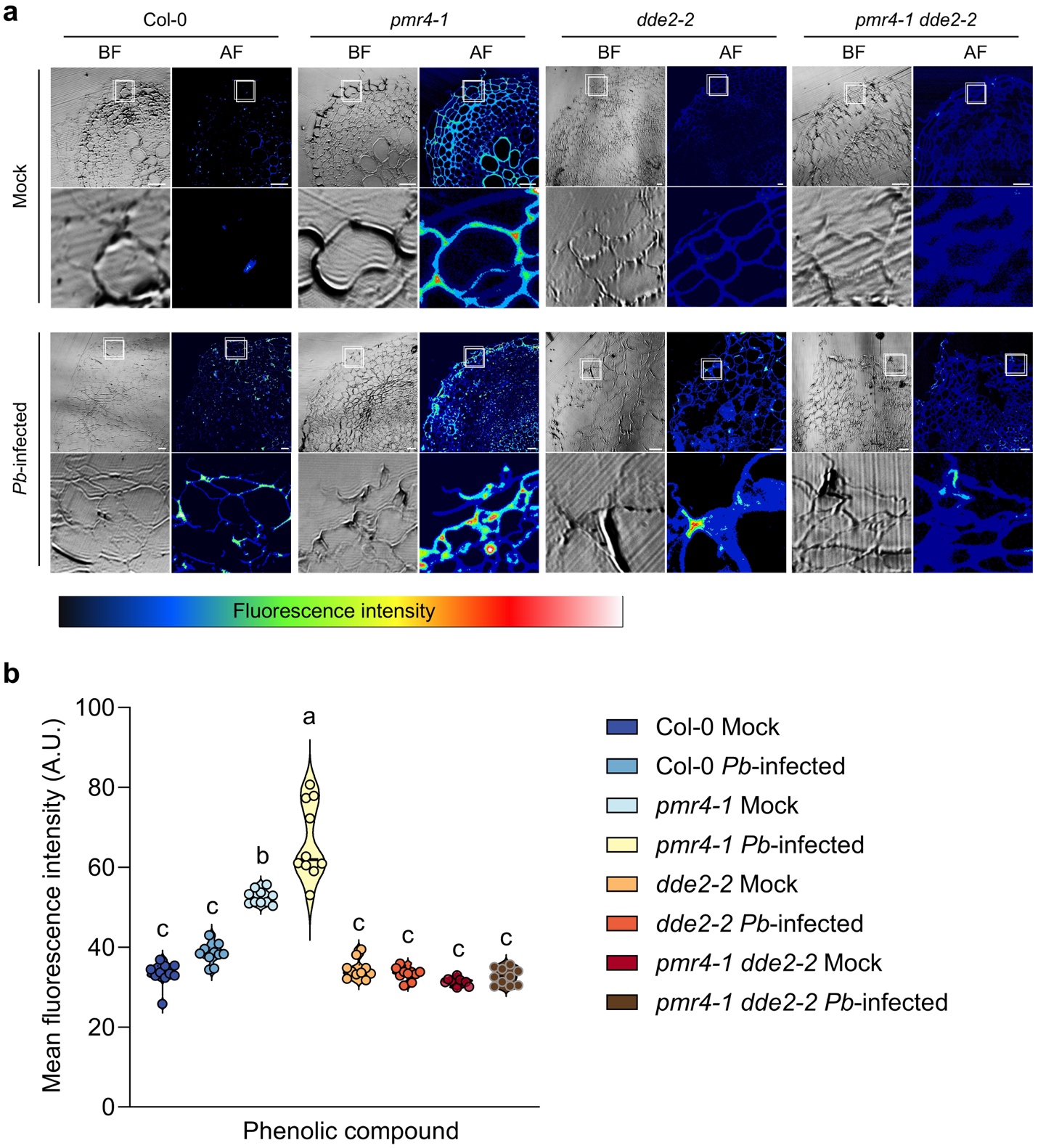
**

Figure S15

**Figure S15.** 405 nm-Autofluorescence-revealed phenolic compounds in mock and *P. brassicae*-infected roots. **a**) Representative images of 405 nm-autofluorescence-based phenolic compound detection. Periderm was labelled with white squares and enlarged in corresponding lower rows. Scale bars = 20 µm. A pseudo-color scheme was applied to visualize the spatial distribution and relative abundance of the detected cell wall components, with colors ranging from dark to bright indicating increasing fluorescence intensity. Root cross sections have a thickness of 0.5 µm. BF, bright field; AF, autofluorescence. **b**) Quantitative analysis of relative phenolic compound deposition levels as observed from (A). Mean fluorescence intensity was analyzed by Fiji software. 10 randomly selected areas from 5 independent roots were calculated and One-way ANOVA was used to show the statistical significance. (α = 0.05). Different letters represent statistical significance. This figure is to provide complementary experimental data as shown in Fig. 7a and b.


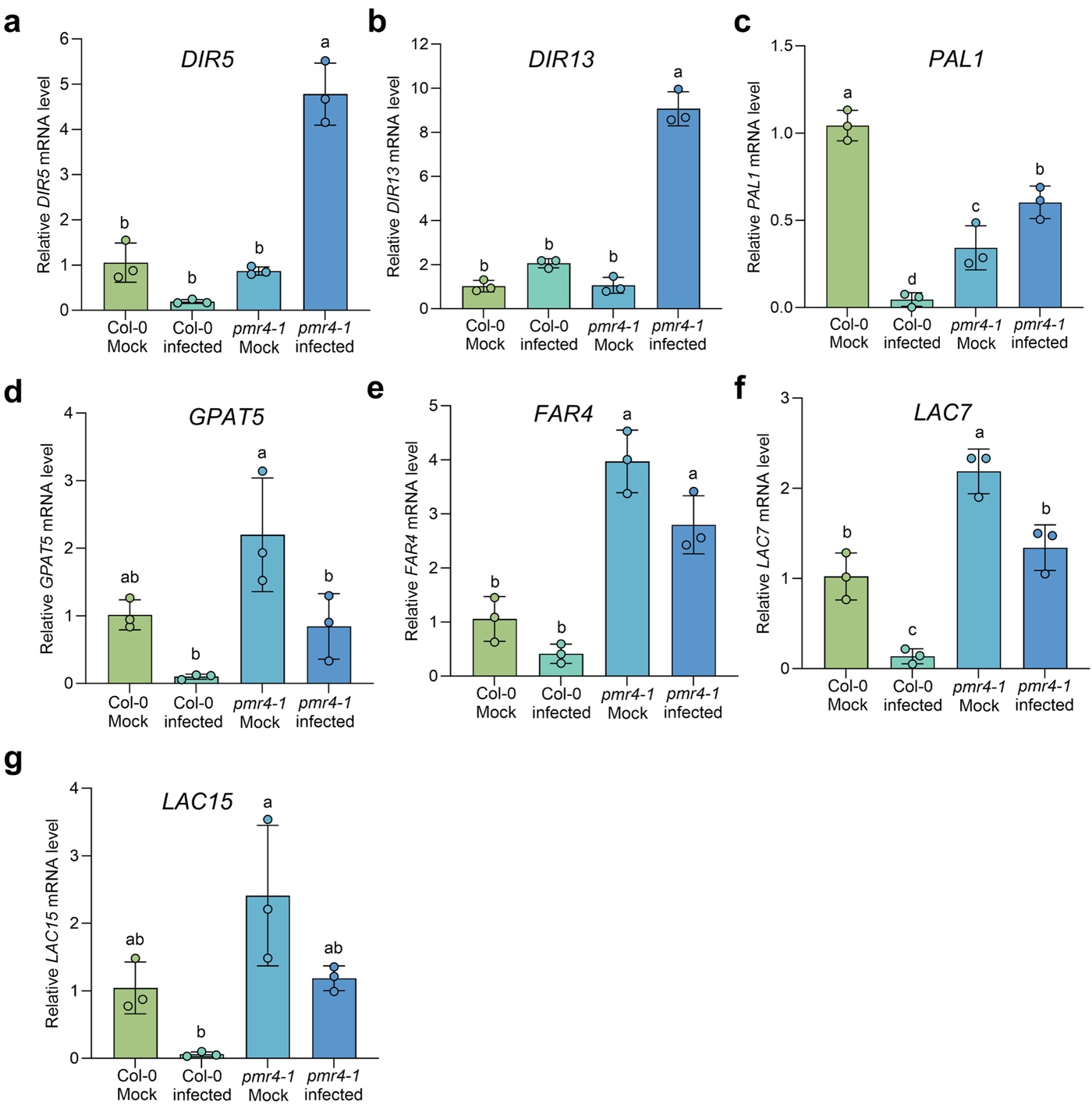


Figure S16

**Figure S16.** RT-qPCR validation of RNA-seq data for genes involved in phenolic polymer assembly, phenylpropanoid synthesis, suberin synthesis and fatty acid synthesis. Relative mRNA level of **a**) *DIR5*; **b**) *DIR13*; **c**) *PAL1*; **d**) *GPAT5*; **e**) *FAR4*; **f**) *LAC7*; and **g**) *LAC15*. cDNA templates were generated from the same group of RNA-seq RNAs. *AtUBQ10* was used as an internal reference gene. The gene expression from mock Col-0 was selected as the control reference when calculating the relative expression profiles. One-way ANOVA was used to show the statistical significance. (α = 0.05). Letters represent significant differences. Data represent means ± SD. n =3. This figure aims to validate Fig. 8c.

**References**

**Krogh, A., Larsson, B., von Heijne, G., and Sonnhammer, E.L.** (2001). Predicting transmembrane protein topology with a hidden Markov model: application to complete genomes. J Mol Biol **305,** 567-580.

**Letunic, I., and Bork, P.** (2021). Interactive Tree Of Life (iTOL) v5: an online tool for phylogenetic tree display and annotation. Nucleic Acids Res **49,** W293-W296.
